# Body weight affects ω-3 polyunsaturated fatty acid (PUFA) accumulation in youth following supplementation in post-hoc analyses of a randomized controlled trial

**DOI:** 10.1371/journal.pone.0173087

**Published:** 2017-04-05

**Authors:** Lisa M. Christian, Andrea S. Young, Amanda M. Mitchell, Martha A. Belury, Barbara L. Gracious, L. Eugene Arnold, Mary A. Fristad

**Affiliations:** 1 Department of Psychiatry and Behavioral Health, The Ohio State University Wexner Medical Center, Columbus, OH, United States of America; 2 The Institute for Behavioral Medicine Research, The Ohio State University Wexner Medical Center, Columbus, OH, United States of America; 3 Department of Obstetrics and Gynecology, The Ohio State University Wexner Medical Center, Columbus, OH, United States of America; 4 Department of Psychology, The Ohio State University, Columbus, OH, United States of America; 5 Department of Psychiatry and Behavioral Sciences, Johns Hopkins University, Baltimore, MD, United States of America; 6 Program of Nutrition in the Department of Human Sciences, The Ohio State University, Columbus, OH, United States of America; 7 The Research Institute at Nationwide Children’s Hospital, Columbus, OH, United States of America; Garvan Institute of Medical Research, AUSTRALIA

## Abstract

Guidelines for suggested intake of ω-3 polyunsaturated fatty acids (PUFAs) are limited in youth and rely primarily on age. However, body weight varies considerably within age classifications. The current analyses examined effects of body weight and body mass index (BMI) on fatty acid accumulation in 64 youth (7–14 years) with a diagnosed mood disorder in a double-blind randomized-controlled trial (2000mg ω-3 supplements or a control capsule) across 12 weeks. Weight and height were measured at the first study visit and EPA and DHA levels were determined using fasting blood samples obtained at both the first and end-of-study visits. In the ω-3 supplementation group, higher baseline body weight predicted less plasma accumulation of both EPA [B = -0.047, (95% CI = -0.077; -0.017), β = -0.54, p = 0.003] and DHA [B = -0.02, (95% CI = -0.034; -0.007), β = -0.52, p = 0.004]. Similarly, higher BMI percentile as well as BMI category (underweight, normal weight, overweight/obese) predicted less accumulation of EPA and DHA (ps≤0.01). Adherence to supplementation was negatively correlated with BMI percentile [B = -0.002 (95% CI = -0.004; 0.00), β = -0.44, p = 0.019], but did not meaningfully affect observed associations. As intended, the control supplement exerted no significant effect on plasma levels of relevant fatty acids regardless of youth body parameters. These data show strong linear relationships of both absolute body weight and BMI percentile with ω-3 PUFA accumulation in youth. A dose-response effect was observed across the BMI spectrum. Given increasing variability in weight within BMI percentile ranges as youth age, dosing based on absolute weight should be considered. Moreover, effects of weight should be incorporated into statistical models in studies examining clinical effects of ω-3 PUFAs in youth as well as adults, as weight-related differences in effects may contribute meaningfully to inconsistencies in the current literature.

**Trial registration.** WHO International Clinical Trial Registry Platform NCT01341925 and NCT01507753

## Introduction

Long-chain ω-3 polyunsaturated fatty acids (PUFAs) play an important role in early life health and development, with potential effects even prior to birth.[1] It is well established that ω-3 PUFAs, particularly docosahexaenoic acid (DHA; 22:6n-3), are critical to fetal growth, as well as neural and retinal development.[2, 3] Though data are mixed, greater maternal ω-3 PUFA consumption has been linked with longer gestation and reduced risk for asthma in offspring.[4–9] Moreover, infant consumption may benefit language, motor, and cognitive development.[10–12]

While the majority of studies on ω-3 PUFAs and development have been conducted in pregnant women and infants/toddlers, observational and experimental data indicate that ω-3 PUFA consumption is beneficial for cardiovascular health in youth (8–15 years of age), as indicated by lower systolic blood pressure and increased high-density lipoprotein (HDL).[13–15] In addition, randomized controlled trials and open-label trials support a beneficial role ω-3 PUFAs in reducing depressive and manic symptoms in youth between age 6 and 17.[16–20]

Available recommendations for youth intake of ω-3 PUFAs rely largely on age.[21–23] Age-related dose increases reflect greater needs due to changes in body weight; between ages 2 and 8, weight increases ~138% and 141% for boys and girls, respectively, with an additional increase of ~110% and 84% in boys and girls from the ages 9 and 18.[24] However, weight at a given age varies considerably.[25] Moreover, ~32% of youth ages 2–19 in the US are overweight, including 16.9% identified as obese. Some data indicate that body mass index (BMI) is a more important predictor of response to dietary supplementation than is simple weight.[26] Thus, data on the role of weight and BMI in fatty acid accumulation in youth would be informative.

The current analyses examined plasma polyunsaturated fatty acids (PUFAs) among 64 children and adolescents ages 7–14 years randomized to receive either ω-3 PUFA supplements or control capsules for 12 weeks. The predictive value of body weight, BMI percentile, and BMI category (underweight, normal weight, overweight/obese) for accumulation of relevant fatty acids was determined.

## Materials and methods

### Participants

**Fig 1:**
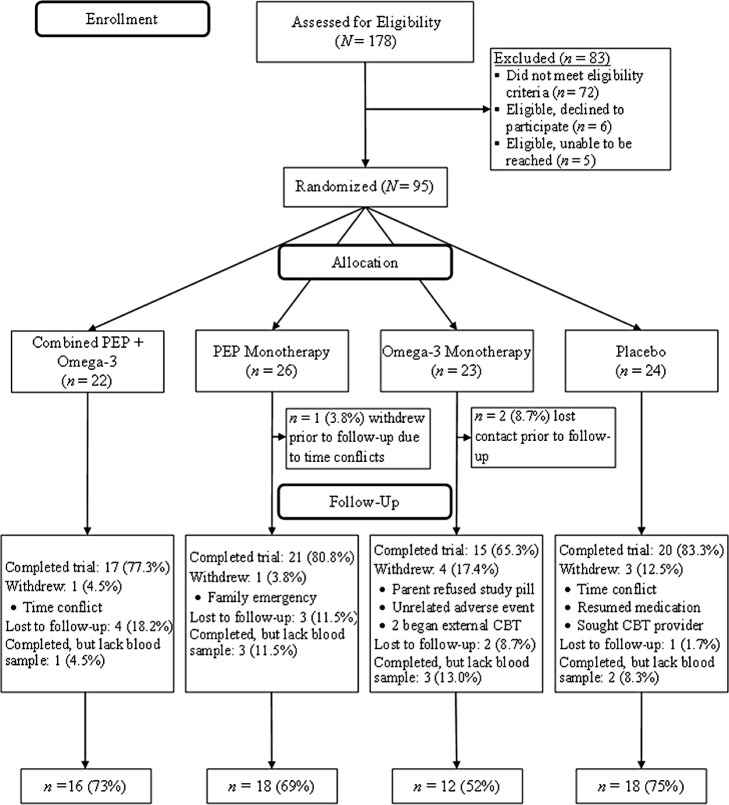
Recruitment and selection flow chart for randomized controlled trial.

This study included 95 youth ages 7–14 years who completed a 12-week NIMH-funded RCT in which they were randomized to receive either ω-3 supplements (n = 45) or control capsules (n = 50). Overall, 64 participants completed the trial and had blood samples available at both time points, resulting in a final analytic sample of 28 in the supplemented group and 36 in the control group. (See Fig 1 for recruitment and selection flow chart).[27] Body parameter measurements and fasting blood samples were collected at the University Medical Center.

The participant sample was obtained from two NIMH-funded R34 studies (ClinicalTrials.gov identifiers: NCT01341925 NCT01507753), the goals of which were to determine feasibility and effects sizes for PUFA supplementation and family-focused, skills-based therapy for mood disorders [28, 29]. Secondary goals were to explore response curves over time, mediators and moderators, treatment response across a broad array of outcome variables, adherence to treatment, and side effects. Given pragmatic limitations (time frame, budget) of an R34, we sought to enroll 60 youth in each trial. One was exceeded by 20%, the other study under-recruited by over 50%. Among those included in the current post-hoc analyses, 57% (n = 16/28) of the supplement group and 50% (n = 18/36) of the control group also received psychotherapy. All procedures were approved by The Ohio State University Biomedical Institutional Review Board and carried out in accordance with relevant guidelines; informed consent and assent were obtained from parents and youth, respectively. Only methods pertinent to the current analyses are described here; further details regarding recruitment and study design can be found elsewhere.[28, 29]

### Ω-3 and control capsules

Families received a pill organizer at each visit containing the ω-3 capsules or control as well as daily multivitamin/mineral tablets. Pill organizers were filled by staff not otherwise involved in the study; all study staff who directly interacted with participants were blinded to ω-3 treatment group assignment. The multivitamin/mineral tablets were included to standardize micronutrient levels across youth. No nutritional supplements other than multivitamin/mineral tablets were permitted the month prior to or during study treatment. Youth randomized to ω-3 received two 500mg ω-3 capsules (350mg EPA, 50mg DHA; 100mg other ω-3) twice daily for a total daily dose of 2000mg of ω-3 (1400mg EPA, 200mg DHA; 400mg other). Those randomized to the control group received capsules matched for odor and appearance to active capsules. The control capsules were comprised primarily of linoleic acid (18:2ω6; 42%), oleic acid (18:1ω7; 41%), and palmitic acid (16:0; 13%). OmegaBrite (www.omegabrite.com; Las Vegas, NV) provided both the ω-3 and control capsules.

### Demographics and anthropometrics

Parents provided information on child race, ethnicity, sex, and date of birth. Weight and height were measured at the first study visit. BMI was calculated as kg/m^2^. Age- and sex-adjusted BMI percentiles were determined per Centers for Disease Control and Prevention (CDC) guidelines.[30] BMI categories per CDC were as follows: underweight: < 5^th^ percentile, normal weight: 5^th^ to < 85^th^ percentile, overweight: 85^th^ to <95^th^ percentile, and obese: ≥ 95^th^ percentile.

### Plasma fatty acid assays

Non-esterified fatty acids were analyzed by gas chromatography of plasma levels of fatty acids using a two-step procedure.[31] Total lipids were extracted using the Bligh and Dyer method.[32] Fatty acid methyl esters (FAMEs) were prepared from each lipid fraction by incubating samples with tetramethylguanidine at 95°C[33] and quantified using a Hewlett Packard 5890 gas chromatograph equipped with an auto-sampler ChemStation software (Agilent Technologies, Meriden, CT) FAME ionization detector and a 30-m Omegawax 320 capillary column (Supelco Co.).[31] Helium flow rate was 30 ml/min and oven temperature was programmed to start at 175°C then ramped to 220°C at 3°C/minute. FAMEs were identified by comparing retention times of samples to retention times of authentic standards (Supelco Co.).

### Adherence

At 2, 4, 6, 9, and 12 week assessments following randomization, each participant received ω-3 supplements or control capsules in a pill organizer. Adherence was assessed from the number of pills remaining in the returned pill organizers at each assessment, and calculated to reflect the percentage of total pills taken out of those provided. Parents were asked to inform study staff of any discarded pills to ensure accurate capsule counts in the returned pill organizers.

### Analytic approach

Presence of change in relevant PUFAs from baseline to 12 weeks of supplementation was determined via repeated measures analysis of variance (ANOVA). For analyses of effect of body parameters on change in PUFAs, change scores were calculated for increases in plasma levels of relevant PUFAs from baseline through 12 weeks of supplementation. Regression analyses were conducted to examine the associations between both body weight and BMI percentile (as measured at the baseline visit) with changes in EPA and DHA status (supplement group) or palmitic acid, oleic acid, and linoleic acid (control group). In addition, one-way analysis of variance (ANOVA) was used to examine changes in plasma PUFA levels in association with BMI categories. When significant differences were observed, post-hoc tests using Fisher’s least significant difference (LSD) test were employed to determine the presence of differences between specific groups. This method does not account for multiple comparisons. The role of adherence was examined using regression analyses and ANCOVA adjusting for adherence per the a priori hypothesis that adherence may co-vary with body weight. All analyses were conducted using SPSS 24.

## Results

### Sample characteristics

Demographic characteristics are summarized in Table 1. Participants were 7–14 years of age and majority White. The sample included youth from a wide range of household incomes (range: < $20,000 to >$80,000). Adherence rates were 88% and 85% in the ω-3 supplement and control capsule groups, respectively. Active supplement and control groups did not differ significantly in baseline levels of EPA [t(62) = 0.34, p = 0.737] or DHA [t(62) = -0.36, p = 0.718].

**Table 1:** Demographic characteristics and capsule adherence.

	Ω3 Group (*n* = 28)		Placebo Group (*n* = 36)	
Characteristic	Frequency (*n*)	Proportion (%)	Frequency (n)	Proportion (%)
Hispanic ethnicity	4	14.3%	2	5.6%
Race				
White race	15	53.6%	25	69.4%
Black	7	25.0%	7	19.4%
Asian	1	3.6%	0	0.0%
Bi/multi-racial	5	17.9%	4	11.1%
Male sex	18	64.3%	23	63.9%
Annual Household Income ^Ɨ^				
< $20,000	7	25.0%	4	11.1%
$20,000–40,000	5	17.9%	9	25.0%
$40,000–60,000	3	10.7%	11	30.6%
$60,000–80,000	5	17.9%	5	13.9%
>$80,000	7	25.0%	7	19.4%
BMI-for-age Classification ^ƗƗ^				
Underweight (< 5^th^ %ile)	7	25.0%	12	33.3%
Healthy weight (5^th^–< 85^th^ %ile)	13	26.4%	10	27.8%
Overweight (85^th^–< 95^th^ %ile)	2	7.2%	5	13.9%
Obese (≥ 95^th^ %ile)	6	21.4%	9	25.0%
**Baseline Characteristic**	**Range**	***M ± SD***	**Range**	***M ± SD***
Child age (years)	7.11–14.61	11.07 ± 2.15	7.44–14.96	11.13 ± 2.40
Body Mass Index (BMI) %ile	1–99	64.14 ± 31.74	1–98	62.17 ± 33.79
Weight (kgs)	22.68–76.57	45.79 ± 16.48	24.49–87.10	44.75 ± 17.30
Pill Adherence (%)	48.26–100.00	87.93 ± 15.18	37.00–100.00	84.96 ± 15.29
Baseline (EPA) levels^Ω^	0.14–0.51	0.30 ± 0.09	0.08–0.51	0.31 ± 0.11
Baseline (DHA) levels ^Ω^	1.31–2.78	2.00 ± 0.45	1.21–3.21	1.96 ± 0.47

^Ɨ^ One participant did not report household income

^ƗƗ^ Per CDC guidelines

^Ω^ mg/100 mg total plasma fatty acids

Among those in the control condition, there was no change in plasma levels of the fatty acids found in the control capsules: linoleic acid (F(1,35) = 0.49, p = 0.49), oleic acid (F(1,35) = 1.44, p = 0.24), or palmitic acid (F(1,35) = 0.09, p = 0.77). Further, those in the control group did not show changes in plasma levels of EPA (F(1,35) = 1.12, p = 0.74) or DHA (F(1,35) = 0.27, p = 0.61). Finally, no significant associations were observed between body weight (kgs), BMI percentile, or BMI classification and change in any of these fats (ps ≥ 0.16). Thus, as intended, the control capsules did not exert a measurable physiological effect regardless of body parameters. Therefore, subsequent analyses focused solely on the ω-3 supplement group.

### Demographic and behavioral correlates in the ω-3 supplement group

Associations among weight, BMI percentile, household income, and adherence to the supplementation protocol in the ω-3 group were examined using linear regression. Adherence was not associated with weight [B = -0.002 (95% CI = -0.006; 0.001), β = -0.24, p = 0.21], but was negatively associated with BMI percentile [B = -0.002 (95% CI = -0.004; 0.00), β = -0.44, p = 0.019]. In addition, higher household income predicted both greater adherence [B = 0.04 (95% CI = 0.011; 0.071), β = 0.49, p = 0.009] and lower BMI percentile [B = -8.05 (95% CI = -14.37; -1.72) β = -0.44, p = 0.019].

### Weight and BMI indicators in association with baseline PUFA levels

Regression analyses showed no associations between body weight and plasma levels of either EPA [B = -0.001 (95% CI = -0.003; 0.001), β = -0.14, p = 0.47] or DHA [B = -0.001 (95% CI = -0.012; 0.01), β = -0.03, p = 0.87] at baseline. Similarly, regression analyses showed no significant associations between age- and sex-adjusted BMI percentile and baseline EPA [B = 0.00 (95% CI = -0.001; 0.002), β = 0.16, p = 0.41] or DHA [B = 0.005 (95% CI = -0.001; 0.01), β = 0.34, p = 0.075], although the latter approached statistical significance. Finally, one-way ANOVA demonstrated no significant differences among participants classified as underweight, normal weight, or overweight/obese in baseline EPA (F(2, 25) = 0.12, p = 0.89) or DHA (F(2,25) = 0.33, p = 0.73).

### Body weight and PUFA accumulation

**Fig 2:**
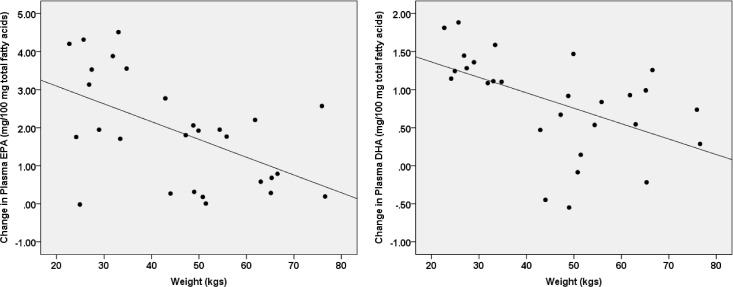
Associations between baseline body weight and plasma PUFA changes. Higher body weight predicted less increase in plasma levels of both EPA [B = -0.047 (95% CI = -0.077; -0.017), β = -0.54, p = 0.003] and DHA [B = -0.02 (95% CI = -0.034; -0.007), β = -0.52, p = 0.004] following supplementation. *Standardized coefficients (β) shown in figure*.

Weight (kg) at the baseline assessment was significantly associated with changes in fatty acid status from baseline to 12 weeks of supplementation for both EPA [B = -0.047 (95% CI = -0.077; -0.017), β = -0.54, p = 0.003] and DHA [B = -0.02 (95% CI = -0.034; -0.007), β = -0.52, p = 0.004; Fig 2]. These associations remained after adjusting for adherence [EPA: B = -0.04 (95% CI = -0.069; -0.011), β = -0.46, p = 0.009; DHA: B = -0.017 (95% CI = -0.029; -0.004), β = -0.43, p = 0.012].

### Age- and sex-adjusted BMI percentiles and PUFA accumulation

Lower BMI percentile was associated with greater increases in plasma EPA [B = -0.21 (95% CI = -0.37; -0.004), β = -0.46, p = 0.014] and DHA [B = -0.013 (95% CI = -0.019; -0.007), β = -0.64, p < 0.001] following supplementation. The association between BMI percentile and EPA accumulation no longer reached statistical significance after adjusting for adherence [B = -0.15 (95% CI = -0.032; 0.003), β = -0.32, p = 0.098], however the association with DHA accumulation remained statistically significant [B = -0.11 (95% CI = -0.017; -0.004), β = -0.53, p = 0.003].

### BMI categorization and PUFA accumulation

Finally, significant differences were observed in EPA accumulation based on BMI category (F(2, 25) = 5.6, p = 0.01). Post-hoc tests showed less EPA accumulation in the overweight/obese group in comparison to the normal weight group (mean difference = -1.12, 95% CI = -2.27, 0.02; p = 0.05) and underweight group (mean difference = -2.14, 95% CI = -3.46, -0.82, p = 0.003). In addition, a trend was observed for lesser EPA accumulation among normal weight versus underweight (mean difference = -1.02; 95% CI = -2.21; 0.18, p = 0.09). ANCOVA analyses demonstrated that this effect was reduced upon adjusting for adherence (F(2,24) = 2.98, p = 0.07).

Similarly, higher BMI (per BMI category) predicted less plasma DHA accumulation (F(2, 27) = 5.8, p = 0.008). ANCOVA analyses indicated that this effect was reduced by adjusting for adherence (F(2,24) = 3.11, p = 0.06). Post-hoc tests showed greater DHA accumulation in underweight versus both normal weight (mean difference = 0.645, 95% CI = 0.11; 1.18, p = 0.02) and overweight/obese groups (mean difference = 0.96, 95% CI = 0.37; 1.55, p = 0.003). However, DHA accumulation among normal weight participants did not differ significantly from that observed in overweight/obese (mean difference = 0.31, 95% CI = -0.20; 0.83, p = 0.219).

## Discussion

In this double blind RCT, increases in plasma PUFAs among supplemented youth were associated with body weight parameters. Specifically, higher weight as well as higher BMI percentile predicted less robust increases in plasma levels of both EPA and DHA. Ranging from r = -0.42 to r = -0.64, these correlations represent large effect sizes. Notably, no differences were observed in baseline plasma DHA or EPA levels based on overall weight or BMI percentile. Thus, observed effects of body parameters on fatty acid accumulation were not accounted for by pre-existing differences in dietary intake or effects of baseline status.

In this cohort, body weight was not associated with adherence to the supplementation protocol. This is not surprising given that body weight is largely a function of age in youth. However, age- and sex-adjusted BMI percentile showed a strong negative correlation with adherence. BMI percentile was also strongly negatively correlated with household income. This is consistent with prior data showing greater rates of obesity and overweight in lower income groups.[34–36] Further, low income has previously been identified as a predictor of poorer medication adherence in youth.[e.g., 37] Associations between BMI percentile and both EPA and DHA accumulation were reduced after inclusion of adherence in the model; however, the magnitude of this reduction did not suggest that this was the primary driver of the association. Thus, in clinical practice, youth from lower socioeconomic backgrounds may experience the combined supplementation impediment of both poorer adherence and higher PUFA intake needs (due to higher rates of overweight/obesity).

In this study, the predictive value of both body weight (kg) as well as age- and sex-adjusted BMI percentile were of interest; while body weight is largely a function of age in those 7–14 years, BMI percentile provides information on the extent to which the current weight meets health targets. Of note, while widely used, the relationship between BMI and actual body composition (i.e., fat versus lean mass) is highly imprecise [38]. However, as the current data showed that both total weight and BMI percentile were highly predictive of plasma EPA and DHA accumulation, it is unlikely that more sophisticated assessment methods such as dual energy x-ray absorptiometry (DXA) to provide lean vs fat mass would add to understanding of this relationship.

From a clinical standpoint, given the increasing variability in weight within a given BMI percentile range as youth age,[39] weight is arguably more universally applicable across the age spectrum to guide clinical dosing. This is particularly true given that the current data do not suggest that clinical categories (such as overweight/obesity) provide additional predictive information about fatty acid accumulation. Rather, linear relationships were observed across the spectrum of weight/BMI percentiles.

Although the optimal PUFA status in children and adolescents is not yet established, the observed effect of weight parameters are likely seen at any given dose. For example, data from 48 women randomized to one of four ω-3 PUFA doses (0.84, 2.52, 5.04 or 7.56 g/day of DHA+EPA) found lower incremental increases in serum and breast adipose tissue levels of both EPA and DHA in relation to higher BMI; this effect did not differ by dose.[40]

From a research standpoint, these data highlight the need to consider effects of weight in observational as well as intervention studies involving ω-3PUFAs. In observational studies, body weight may moderate effects; clinical benefits may be less apparent in heavier youth thereby masking effects in the overall group. In supplementation trials in children as well as adults, dosage by weight should be considered if the trial aims for equivalent biological effects across the study sample.

A strength of this study is that it was conducted as a double-blind, randomized controlled trial. However, it is not without limitations. In the current study, we examined plasma fatty acid levels. In children with a higher BMI, there is a possibility of a larger blood volume diluting values for biomarkers including fatty acids. At this time, we are not aware of evidence documenting this effect in children. However, we cannot eliminate it as a possible confounding factor to explain lower concentrations of fatty acids in larger children. Moreover, plasma measurements may reflect recent fat intake. Erythrocyte fatty acid composition is less likely to fluctuate due to day to day variation of fatty acid intake compared to plasma fatty acid analyses [41]. Although erythrocyte fatty acid composition is the ideal biomarker for habitual dietary fat intake, plasma fatty acid composition is a fairly robust indication of intake, especially if one assumes that most people do not vary their fat intake patterns significantly. In addition, samples were taken in a fasting state. However, in future studies, the utilization of erythrocyte fatty acid composition should be considered.

In sum, the current study demonstrates clear linear relationships of both body weight and BMI with plasma ω-3 PUFA accumulation in children and adolescents. These data did not show that clinical overweight or obesity were particularly predictive; rather, a dose-response effect was observed across the BMI spectrum. Guidelines for optimal ω-3 PUFA intake in youth should consider using weight rather than age-based determinations. Studies examining potential clinical effects of ω-3 PUFAs in youth as well as adults should incorporate the effects of weight into statistical models, as weight-related differences in effects may contribute meaningfully to inconsistencies in the current literature.

## Supporting information

S1 CONSORT Checklist (DOC)

S1 Trial protocol Bipolar disorder (BD). (PDF)

S2 Trial protocol Major depressive disorder (MDD) and dysthymic disorder (DD). (PDF)

S1 Dataset (XLS)
