# Supplementary material for: Body weight affects ω-3 polyunsaturated fatty acid (PUFA) accumulation in youth following supplementation in post-hoc analyses of a randomized controlled trial
Source: PLoS One. 2017 Apr 5;12(4):e0173087. doi: 10.1371/journal.pone.0173087 (PMC5381773; doi:10.1371/journal.pone.0173087)
Supplement: S1 CONSORT Checklist — (DOC) [file pone.0173087.s001.doc]

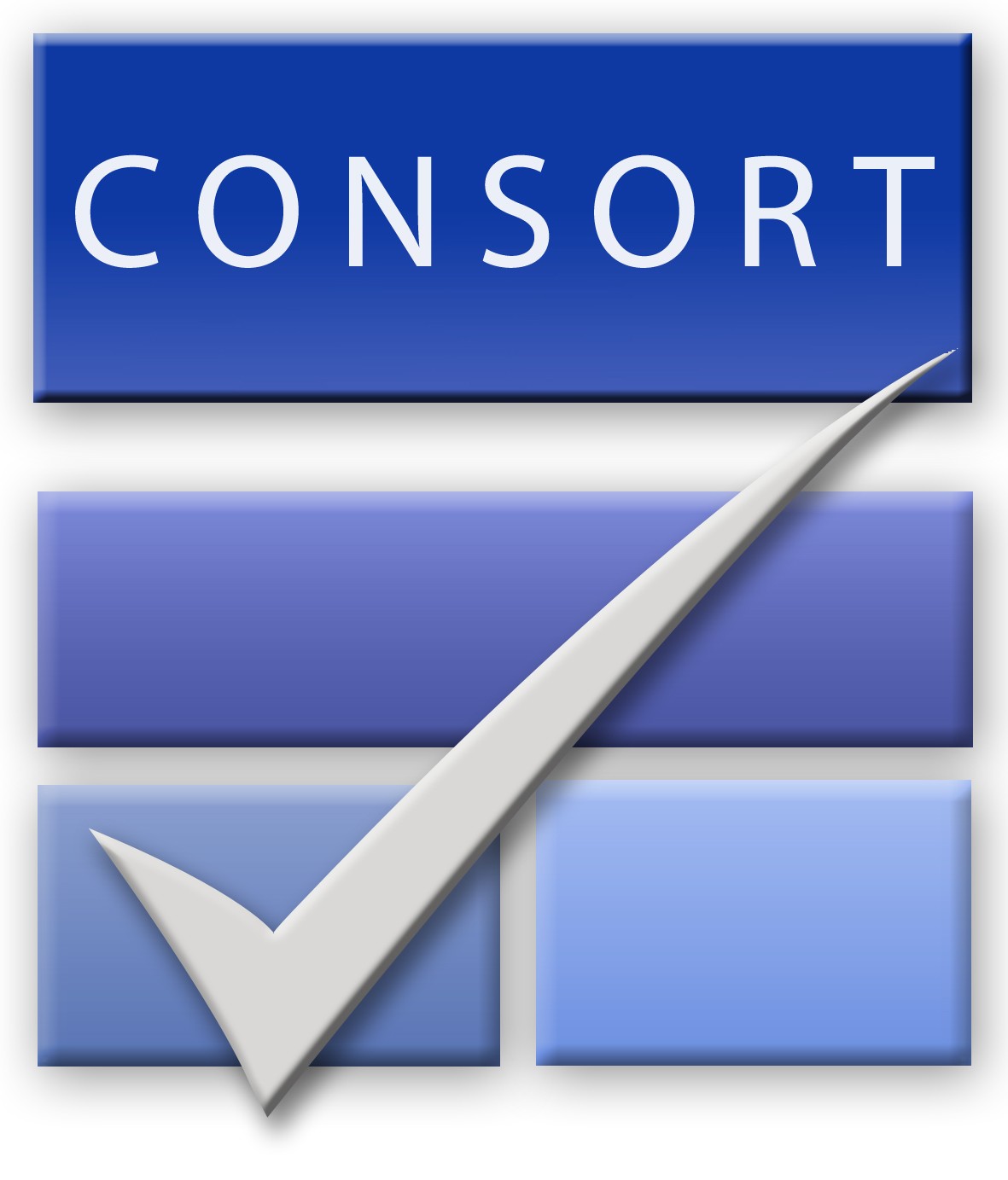
CONSORT 2010 checklist of information to include when reporting a randomised trial*

| Section/Topic | Item No | Checklist item | Reported on page/line No |
| --- | --- | --- | --- |
| Title and abstract | | | |
|  | 1a | Identification as a randomised trial in the title | Page 1 |
|  | 1b | Structured summary of trial design, methods, results, and conclusions (for specific guidance see CONSORT for abstracts) | Page 2 |
| Introduction | | | |
| Background and objectives | 2a | Scientific background and explanation of rationale | Pages 3 & 4 |
|  | 2b | Specific objectives or hypotheses | Line 45-49 |
| Methods | | | |
| Trial design | 3a | Description of trial design (such as parallel, factorial) including allocation ratio | Lines 52-57 |
|  | 3b | Important changes to methods after trial commencement (such as eligibility criteria), with reasons | Supp. Figure 1 |
| Participants | 4a | Eligibility criteria for participants | Lines 52-54 & 64-65 |
|  | 4b | Settings and locations where the data were collected | Lines 56-57 |
| Interventions | 5 | The interventions for each group with sufficient details to allow replication, including how and when they were actually administered | Lines 66-78 |
| Outcomes | 6a | Completely defined pre-specified primary and secondary outcome measures, including how and when they were assessed **(of this specific study)** | Lines 80-96 |
|  | 6b | Any changes to trial outcomes after the trial commenced, with reasons | NA |
| Sample size | 7a | How sample size was determined | Lines 64-65 |
|  | 7b | When applicable, explanation of any interim analyses and stopping guidelines | NA |
| Randomisation: |  |  |  |
| Sequence generation | 8a | Method used to generate the random allocation sequence | Lines 64-65 |
|  | 8b | Type of randomisation; details of any restriction (such as blocking and block size) | Lines 64-65 |
| Allocation concealment mechanism | 9 | Mechanism used to implement the random allocation sequence (such as sequentially numbered containers), describing any steps taken to conceal the sequence until interventions were assigned | Lines 64-65 |
| Implementation | 10 | Who generated the random allocation sequence, who enrolled participants, and who assigned participants to interventions | Lines 64-65 |
| Blinding | 11a | If done, who was blinded after assignment to interventions (for example, participants, care providers, those assessing outcomes) and how | Lines 64-65 & 67-70 |
|  | 11b | If relevant, description of the similarity of interventions | Lines 74-78 |
| Statistical methods | 12a | Statistical methods used to compare groups for primary and secondary outcomes **(of this specific study)** | Lines 103-113 |
|  | 12b | Methods for additional analyses, such as subgroup analyses and adjusted analyses | NA |
| Results | | | |
| Participant flow (a diagram is strongly recommended) | 13a | For each group, the numbers of participants who were randomly assigned, received intended treatment, and were analysed for the primary outcome | Supp. Figure 1 |
|  | 13b | For each group, losses and exclusions after randomisation, together with reasons | Supp. Figure 1 |
| Recruitment | 14a | Dates defining the periods of recruitment and follow-up | Lines 64-65 |
|  | 14b | Why the trial ended or was stopped | Lines 64-65 |
| Baseline data | 15 | A table showing baseline demographic and clinical characteristics for each group | Table 1 |
| Numbers analysed | 16 | For each group, number of participants (denominator) included in each analysis and whether the analysis was by original assigned groups | Lines 52-56 |
| Outcomes and estimation | 17a | For each primary and secondary outcome, results for each group, and the estimated effect size and its precision (such as 95% confidence interval) **(of this specific study)** | Lines 122-163; Fig. 1 |
|  | 17b | For binary outcomes, presentation of both absolute and relative effect sizes is recommended | NA |
| Ancillary analyses | 18 | Results of any other analyses performed, including subgroup analyses and adjusted analyses, distinguishing pre-specified from exploratory | NA |
| Harms | 19 | All important harms or unintended effects in each group (for specific guidance see CONSORT for harms) | Lines 64-65 |
| Discussion | | | |
| Limitations | 20 | Trial limitations, addressing sources of potential bias, imprecision, and, if relevant, multiplicity of analyses | Lines 64-65 |
| Generalisability | 21 | Generalisability (external validity, applicability) of the trial findings | Lines 189-194;  Lines 200-205 |
| Interpretation | 22 | Interpretation consistent with results, balancing benefits and harms, and considering other relevant evidence | Lines 172-188 |
| Other information | | |  |
| Registration | 23 | Registration number and name of trial registry | Line 253-255 |
| Protocol | 24 | Where the full trial protocol can be accessed, if available | Supp. Documents |
| Funding | 25 | Sources of funding and other support (such as supply of drugs), role of funders | Lines 224-233 |

*We strongly recommend reading this statement in conjunction with the CONSORT 2010 Explanation and Elaboration for important clarifications on all the items. If relevant, we also recommend reading CONSORT extensions for cluster randomised trials, non-inferiority and equivalence trials, non-pharmacological treatments, herbal interventions, and pragmatic trials. Additional extensions are forthcoming: for those and for up to date references relevant to this checklist, see [www.consort-statement.org](http://www.consort-statement.org/).
