# Supplementary material for: Body weight affects ω-3 polyunsaturated fatty acid (PUFA) accumulation in youth following supplementation in post-hoc analyses of a randomized controlled trial
Source: PLoS One. 2017 Apr 5;12(4):e0173087. doi: 10.1371/journal.pone.0173087 (PMC5381773; doi:10.1371/journal.pone.0173087)
Supplement: S1 Trial protocol — (PDF) [file pone.0173087.s002.pdf]

RESEARCH STRATEGY

Significance

Originally considered to be a milder version of bipolar disorder (BD), research now indicates bipolar disorder- not otherwise specified (BP-NOS) is a highly impairing condition, as reviewed below. Considerable gains have been made recently in understanding BP-NOS, in large part by research utilizing clear operational definitions for BP-NOS (cf. the NIMH-funded Course and Outcome of Bipolar Youth [COBY] and Longitudinal Assessment of Manic Symptoms [LAMS] studies). However, clinical trials have focused on youth with Bipolar Disorder- Type I (BP1). No clinical guidelines exist for the treatment of BP-NOS.

BP-NOS Findings

COBY followed a large group of children and adolescents with bipolar spectrum disorders (BPSD), including 153 participants diagnosed with BP-NOS. Of these, 3% were one symptom short of reaching DSM-IV-TR criteria for BP1 or BP2, 12% failed to meet BP2 criteria because they had not had a full major depressive episode. The remaining 85% had an adequate number of symptoms with documented impairment to be diagnosed with BP1 or BP2, but did not have the duration of symptoms for either diagnosis. While youth with BP-NOS were hypothesized to be less impaired than their BP1 and BP2 counterparts, results indicated the three groups did not differ in age of onset, number of years they had experienced manic or depressive symptoms, severity of their worst week of manic and depressive symptoms, comorbidities (except anxiety disorders, which were higher in participants with BP2 than BP-NOS or BP1), suicidal ideation, and family history of mental illness[1]. Participants with BP-NOS were significantly: less likely than those with BP2 to have a suicide attempt in their family; less impaired than participants with BP1, but similar to those with BP2 in terms of functional impairment, suicide attempts, psychiatric hospitalization, and psychopharmacological treatment [1].

At 2-year follow-up, 60% of the original sample was available for study. Over half were diagnosed with BP1, 8% with BP2, and 35% with BP-NOS [2]. Groups remained comparable on comorbid diagnoses, demographics and family history [2]. Participants with BP-NOS were less likely than those with BP1 to have had a lifetime diagnosis of psychosis, although they were similar on weekly reported symptoms of psychosis. While, according to DSM-IV-TR, psychotic symptoms automatically upgrade the diagnosis to BP1, this practice was not followed in COBY when psychosis occurred only within periods of depression; this procedure allowed for more explicit tracking of the course of manic illness. Although recovery rates did not differ, participants with BP-NOS took approximately 3 times as long to recover from their index episode [2]. Of note, the COBY BP-NOS definition did not exclude cases that would meet DSM criteria for cyclothymic disorder, which would also have a longer episode duration characterized by pervasive, low- or moderate-grade mood disturbance. Participants with BP-NOS were less likely to experience a recurrence than those with BP2 [2]. However, when weekly symptom status was compared, the three groups did not differ in percentage of time spent asymptomatic; participants with BP-NOS were significantly more likely to have subsyndromal symptom status and changed symptom status (e.g., asymptomatic to manic, depressed to manic, depressed to subsyndromal) more often than the other two groups. Approximately 30% of participants with BP-NOS converted to BP1 or BP2 by 24-months. Taken together, findings suggest that BP-NOS has unique characteristics of longer time to recovery and recurrence, frequently shifting symptom status, and more time spent with subsyndromal symptoms but not asymptomatic, when compared with BP1 and BP2.

Psychotherapy for BP-NOS

Previous research provides support for the efficacy of various adjunctive individual and family psychoeducational interventions for children aged 12 and under with BPSD, but studies have not been conducted specifically with BP-NOS [3,4,5]. Work in this area is described in greater detail in the *Preliminary Studies* section.

Psychopharmacology for BP-NOS

No pharmacologic treatment guidelines exist for BP-NOS. Available evidence-based pharmacotherapy guidelines are for BP1; efficacious medications are, unfortunately, associated with significant risk for adverse events [6,7]. Additionally, while anti-manic agents have been identified, no study has demonstrated an effective anti-depressant agent for youth with bipolar depression. In a study monitoring side effects of atypical neuroleptics including clozapine, olanzapine, and risperidone, all three caused drowsiness and decreased motor activity; 30-60% of youth taking clozapine experienced constipation, increased salivation, orthostatic hypotension, and nasal congestion. Side effects were seen in patients taking olanzapine and risperidone less often, but 5%-15% of participants taking olanzapine or risperidone suffered from rigidity, tremor, and dystonia. All participants gained weight during the study; those in the olanzapine group gained significantly more weight than those in the other two treatment groups (4.6 ± 1.9 kg; [8]).

Correll [9] reviewed weight gain and metabolic side effects of mood stabilizers and antipsychotic medications

in 19 studies of pediatric bipolar patients. Trials examined lithium, antiepileptics, a combination of these, and second-generation antipsychotics alone or with lithium or divalproex. Significant and clinically relevant weight increases were found in 18 (75%) trials. Weight gain was greater for participants receiving second-generation antipsychotics with mood stabilizers compared to those receiving a single mood stabilizer or multiple mood stabilizers, but not for those taking a single antipsychotic.

Recent clinical trials of depression and bipolar disorders in children and adolescents show approximately 20%-25% of participants dropped out of short-term psychotropic medication treatment trials[10,11]. DelBello and colleagues [11] reported a 25% drop out rate in a single-blind, 12 week study of quetiapine in adolescents age 12-18. Biederman and colleagues[10] conducted an 8 week, open-label trial of aripiprazole in children age 6-17 with bipolar disorder in which they observed a 21% drop out rate. Additionally, a recent study of an anticonvulsant mood stabilizer in children failed to show any superiority to placebo [12]. Previous research suggests omega-3 (Ω3) fatty acids have a beneficial effect on mood with little evidence of negative side-effects or deleterious drug interactions, suggesting Ω3 might function as either a primary or adjunctive treatment with a more favorable risk-benefit ratio for children suffering from BP than currently available pharmacologic interventions[13].

Omega-3 Fatty Acids (Ω3) and Mental Health

Epidemiological studies comparing rates of mood disorders in countries with varied rates of fish consumption **found significant negative correlations ($r = -.84$, $p<.005$[14] & $r = -.74$, $p<.0001$[15]). DHA supplementation in boys has been found to increase activity in attention networks in the prefrontal cortex during sustained attention tasks.[16]** Owen **et al[17]** posited four pathways for Ω3 depletion that may be linked to depression. First, ratios of DHA to other fatty acids affect membrane fluidity and **enzyme** functioning, ion channels, and receptor binding. Second, Ω3 concentrations impact neuroplasticity and cell survival. Third, Ω3 concentrations affect gene expression. Fourth, Ω3 availability **suppresses** production of proinflammatory cytokines, which are elevated in depressed patients and inhibited by some antidepressants.[17]High resolution structural MRIs **show** people with higher intake levels of Ω3 have greater grey matter volume in the anterior cingulate cortex, the right hippocampus, and the right amygdala. These brain areas **are** involved in emotional arousal and emotion regulation **and are** reduced in people with mood disorders.[18] **Others** have demonstrated lowered T_2 values, suggestive of increased membrane fluidity, in patients with bipolar disorder who take Ω3.[19]

Ω3 may improve symptoms in a variety of psychiatric disorders including mood disorders (see Appendix A for summary of studies). **Pediatric studies show benefits for hyperactivity and stereotypy in autism [20], improvements in inattention, hyperactivity, oppositional/defiant behavior, and conduct problems in children with ADHD [21,22] and improvements in reading, spelling, and behavior in children with developmental coordination disorder [23]. A recent 12-week RCT of 81 individuals, age 13-25, with "ultra-high risk" of psychotic disorder was followed by a 40-week monitoring period.[24] Two of 41 participants in the Ω3 group (4.9%) and 11 of 40 in the placebo group (27.5%) developed psychotic disorder. Compared to the placebo group, those in the Ω3 group showed significantly lower levels of positive symptoms (p = 0.01), negative symptoms (p = 0.02), and general symptoms (p = 0.01) and displayed improved functioning (p = 0.002).[24]**In addition to **safety**, Ω3 may actually provide multiple health benefits.[25,26] **Besides** decreasing psychiatric symptoms,[27,28] Ω3 have been shown to significantly improve cardiovascular and metabolic health and decrease body fat, both independently and in combination with regular exercise.[29]**The latter** is particularly important, as **many** current treatments for mood stabilization are associated with significant weight gain, obesity and metabolic disorders.

Adult mood disorder studies. **Studies conducted to date on Ω3 in the treatment of mood disorders in adults are summarized in Appendix A.** Although available studies are heterogeneous **in methodology and results, a meta-analysis showed** a significant antidepressant effect of Ω3 in the overall sample (N=329, ES=0.61, p=.003), in patients with clearly defined depression (n=222, ES=0.69, p=.002) and bipolar disorder (n=105, ES=0.69, p =.0009).[30]

*Child **mood** studies.* **One RCT in depressed children and one RCT and two open-label trials in youth with BPSD have been reported. A 2006 RCT** examined Ω3 in 28 children aged 6 to 12 with MDD. Participants were randomized to Ω3 (2:1 EPA: DHA ratio, 380-400 mg EPA and 180-200 DHA) or placebo **for 16 weeks**. Eight children dropped out: 5 for noncompliance, 1 for a manic episode, 1 for an endocrine workup, and 1 for nonresponse. No clinically significant side effects were reported. Results **in the 20 completers** demonstrated highly significant effects of Ω3 on depressive symptoms. Seven of ten children who received Ω3 had >50% reduction in depressive symptoms compared to no children in the placebo group. Four of ten children receiving Ω3 met remission criteria; **none of 10 in the placebo group did.** Most of the treatment response noted at week 16 had been achieved by week 12. The lack of clinically relevant side effects reported by participants suggests Ω3 may have a better risk: benefit ratio than traditional anti-depressants.[31]

A 6-week open trial of 360 mg EPA and 1560mg DHA daily in 18 youth with BP1, BP2 or BP-NOS found Ω3 red blood cell levels and levels of EPA and DHA increased significantly from pre- to post-supplementation. Clinician ratings of depression and mania decreased significantly (*p* =.002; *p* =.004) and global functioning increased significantly (*p*<.001). Parent-rated internalizing and externalizing behaviors decreased significantly (*p* =.009; *p* =.014).[32] Another research group conducted an 8-week open trial of Ω3 (7:1 EPA:DHA ratio) as monotherapy (with stimulants allowed) for 20 outpatients aged 6-17 with bipolar disorder.[33] Participants received 1.3 to 4.3 g of Ω3 daily with most (85%) taking ≥2.0 g/day. **Four dropped out for** lack of efficacy, not adverse events. Ω3 caused few mild side effects, mostly gastrointestinal. By Week 8, mania symptoms decreased by 30% in 50% of participants and decreased by 50% in 35% of participants. The 17 who took ≥2.0g of Ω3 fatty acids per day showed greater improvements than those who took <2.0 g per day. Depression ratings also decreased significantly **showing a 10.4 ± 9.9 point reduction on the Children's Depression Rating Scale (CDRS; *p*=0.002)**. By study end, 40% of participants were rated much or very much improved for depression and mania. The authors suggest dosing studies would benefit this body of research, especially in pediatric samples[33]

Lastly, a RCT of flax-seed oil (alpha-linolenic acid [ALA]) vs. olive oil in 44 youth aged 6-17 with BP1 or BP2 found groups did not differ on clinician rated depression and mania symptoms or global functioning. Gracious hypothesized low conversion from alpha-linolenic acid to EPA and DHA, which emphasizes the importance of studying EPA and DHA directly.[34]

Combination Treatment

A series of studies have indicated combination treatment is advantageous in treating anxiety (Child-Adolescent Anxiety Multimodal Study [CAMS][35]), adolescent depression (TADS [36,37]), pervasive developmental disorders[38], and ADHD (Multimodal Treatment Study of Children with ADHD [MTA][39,40,41,42]). Combination treatment has not been examined in children with BP-NOS.

Innovation

Practice parameters for treatment of bipolar disorder in youth[43] state: "a multimodal treatment plan, combining medications with psychotherapeutic interventions, is needed to address the symptomatology and confounding psychosocial factors in children and adolescents with bipolar disorder." The current study will examine efficacy of omega3 and psychotherapy (Individual Family Psychoeducational Psychotherapy [IF-PEP],], a manualized, empirically evaluated treatment), alone and in combination. IF-PEP and its variation, Multi-Family PEP (MF-PEP), have been developed and studied by our research team (see Preliminary Studies). IF-PEP utilizes a biopsychosocial understanding of mood disorders and incorporates cognitive-behavioral and family systems techniques in its interventions. IF-PEP provides psychoeducation, support, and skills development. Parents and children receive workbooks and families receive project handouts at the end of each session. Sessions begin with a brief "check-in" meeting of parents and children, during which the previous week's projects are discussed. Children and parents alternate attending sessions.

Summary

BP-NOS is highly impairing. Despite this, there are essentially no psychopharmacologic guidelines for children with BP-NOS. Medications demonstrated to be efficacious for BP1 carry with them significant risk for adverse events [6,7,44]. Additionally, very little is known about treating the depressive phase of the illness. Psychotherapy trials have indicated adjunctive benefits of psychoeducationally oriented interventions, including psychoeducational psychotherapy (PEP), but no trials to date have focused on children with BP-NOS. Limited studies have been conducted with Ω3 polyunsaturated fatty acids. Clinical trials suggest some benefit for depression, irritability and mood stability; however, double-blind placebo controlled trials are scanty, particularly in children. Of particular interest, Ω3 provides health benefits in the very areas that are problematic for psychotropic medications used to treat BPSD, in particular, weight gain and metabolic disorders. There is considerable question about optimal EPA:DHA ratio and dosing, with some hint of a u-shaped or at least asymptotic dose-response curve and perhaps differential optimal dose for depressive and manic symptoms. Further investigation in controlled clinical trials is needed to examine the safety and benefits of Ω3 as well as PEP in the treatment of childhood BP-NOS. This application proposes to lay the groundwork for undertaking a large randomized clinical trial by testing feasibility, estimating effect sizes and examining a range of potential health outcomes in a pilot study of Ω3 and PEP for children with BP-NOS.

Approach

We have assembled a team with specific expertise in the areas relevant to the application. Dr. Fristad has successfully recruited children (ahead of schedule and over proposed census) in the age range of the proposed study who are diagnosed with mood disorders for studies of adjunctive psychosocial interventions or phenomenologic studies. She has retained the majority of these children and their families up to 54-months

and for 10 to 20 visits, depending on the study. **She has experience with Ω3 in her clinical work.** Dr. Arnold has successfully conducted trials of nutritional interventions for school-aged children diagnosed with ADHD **(including a study of essential fatty acids)** with notable retention rates over Phase 1 and Phase 2 trials **and has experience with clinical use of Ω3**. Dr. Belury is a human nutritionist internationally known for her research with PUFA to influence various medical conditions.[45,46] She is currently working as a Co-Principal Investigator with Jan Kiecolt-Glaser, PhD, another OSU investigator, on a study of omega-3, inflammation and stress in adults. In the current application, Dr. Belury will oversee the examination of how dietary fatty acids influence fatty acid compositions of plasma in children. Mr. Votolato is a highly experienced clinical research pharmacist with extensive collaborative experience in running adult and child mood disorder clinical trials. Dr. Gardner is a developmental-quantitative psychologist who has contributed significantly to the pediatric mental health literature. His training includes a master's degree in statistics and doctorate in psychology.

Preliminary Studies

Over the past 17 years, our research group has initiated a series of efforts to develop and empirically validate MF-PEP and IF-PEP as effective treatment modalities for families of children with mood disorders. These efforts have included: a) identifying developmental adaptations required to modify adult-based programs for use with families of mood disordered children or adolescents;[47] b) developing specific therapeutic techniques to work with young children;[48,49] c) developing manual-based, multi-family psychoeducational group psychotherapy and individual-family psychoeducational psychotherapy programs for families of children with mood disorders;[50]d) developing and testing instruments required to assess variables of interest to our work;[51,52,53,54,55,] e) implementing initial outcome studies;[56,57] f) implementing three RCTs–a pilot study (*N*=35) for MF-PEP [58,59,60] and for PEP (*N*=20)[4] and a large scale (*N*=165) randomized controlled study on MF-PEP (R01 MH61512);[3,60,61] g) trialing the 24-session version of PEP in two children with bipolar disorder[62] h) publishing a treatment manual and workbooks; and i) conducting a pilot efficacy-to-effectiveness trial. We discuss our three RCTs in further detail below.

Small Scale MF-PEP RCT: The Ohio Department of Mental Health (ODMH) Study. We studied 35 children, aged 8-11, with major mood disorders (55% depressive spectrum, 45%, bipolar spectrum) and 47 of their parents (14 fathers, 33 mothers).[58,3,50 47] All were encouraged to continue treatment-as-usual (TAU); 18 were enrolled immediately into a six-session MF-PEP (IMM+TAU) condition; 17 families were enrolled into a 6 month waitlist condition (WLC+TAU), after which they received MF-PEP. Four assessments occurred: Time 1 (T1) at baseline; T2, 2 months post-enrollment (post-MF-PEP for IMM); T3, 6 months post--enrollment (pre-MF-PEP for WLC); and, T4, for the WLC only, post-MF-PEP. Treatment resulted in: 1) increased understanding of mood disorders; 2) improved family interactions; 3) improved consumers of care; 4) increased perceived social support, skill acquisition and attitudinal changes; 5) high degrees of satisfaction with treatment.

Large Scale MF-PEP RCT: The National Institute of Mental Health (NIMH) Study (R01-MH61512). We conducted an efficacy trial of MF-PEP in 165 children aged 8-11 with mood disorders and their primary and secondary caretakers.[60,3,61]Half the families were randomized into immediate treatment (IMM=MF-PEP+TAU); half into a 1 year wait-list condition (WLC= WLC + TAU). After 1 year, WLC families participated in MF-PEP. Assessments occurred at baseline (Time 1, T1), 6 months (T2), 12 months (T3), and 18 months (T4). Treatment resulted in: 1) decreased mood severity; 2) increased understanding of mood disorders; 3) improved consumers of care; 3) high degrees of satisfaction with treatment.

Small Scale IF-PEP RCT: The Ohio Department of Mental Health (ODMH) Study. Pragmatic issues regarding MF-PEP led us to develop a second form of intervention, Individual Family Psychoeducational Psychotherapy (PEP). These issues include: First, many community-based clinicians, especially those in private practice, do not work in settings conducive to leading groups, their case load at any given time would not support running a group for children of similar ages with the same diagnosis. Second, some families may not feel comfortable sharing personal information in a group setting and may prefer more privacy to work on their clinical issues. Third, if clinics offer MF-PEP only once or twice a year, a family with a newly diagnosed child may not wish to wait that long for immediate, tailor-made assistance. Finally, practical issues of how to bill for multiple therapists providing simultaneous service can be a barrier to treatment implementation.

We conducted a pilot study examining IF-PEP in 20 children with BPSD and their parents.[4] Methodology was very similar to the MF-PEP NIMH study, although the assessment was less extensive and data were collected only from the child and a primary parental informant (all mothers). Ten families were randomized to immediate PEP+TAU (IMM); 10 families to a 12-month WLC+TAU (WLC). After one-year, WLC families participated in PEP. Four assessments occurred over 18-months: baseline (Time 1, T1), 6 months (T2), 12 months (T3), and 18 months (T4). PEP session content and projects were nearly identical to that of MF-PEP. However, PEP treatment was delivered in sixteen 50-minute sessions, alternating between parent-only ses-

sions and, after the requisite "check-in," child-only sessions. Fifteen sessions dealt with specific issues associated with BPD. One "in the bank" session was available for use at anytime for families to deal with crises and/or review previous material. The MF-PEP "group games" treatment component was deleted, but a unit on "Healthy Habits" was added that focused on improving sleep hygiene, diet and exercise. This was done because: impaired sleep can trigger a manic episode;[63] exercise can decrease depression;[64] and many medications used to treat BPD are associated with significant weight gain, with nutritional and behavioral alterations needed to combat this adverse side effect.[6]Therapists included two clinical psychology post-doctoral study coordinators and one clinical psychology doctoral candidate who also served as a parent advocate for the Child & Adolescent Bipolar Foundation (CABF). Treatment results included: 1) decreased mood severity (effect size==.45 at six months; effect size=.60 at 12 months); 2) high degrees of consumer satisfaction.

Treatment refinement. Consumer comments, supervision notes and a decision to equalize clinician: family face-to-face time between MF-PEP and IF-PEP led to expanding IF-PEP from 16 to 24 sessions. Five of the additional eight sessions cover: healthy habits (2); school-based issues (1); systems of care (1); and sibling relations (1). The remaining three sessions are additional "in-the-bank" sessions (increasing these from 1 to 4), which can be used as needed by the families to deal with crises and/or review previous material.

Case series trial of PEP 24. This expanded version of PEP was trialed with two families, both of whom showed improvement following treatment. Three out of four mood scales improved from baseline to post-treatment, and parental report of the child's behavior toward family members demonstrated improvement (more positive and less negative interactions following treatment).[62]

Commercialization project. A small business grant (R41 MH077358) was awarded to the Co-PI (MAF/ Child & Family Psychological Services, Inc.) to develop a commercially available treatment manual for PEP. The treatment manual is currently in press. Parent and child PEP workbooks and parent, child and child therapist MF-PEP workbooks are also in press[65]

Design and Method

Sixty children aged 8-12 years with BP-NOS will be randomized to a 2 x 2, **12**-week supplement double-blinded comparison of omega-3 (Ω3) fatty acids vs. placebo (PBO) and psychoeducational psychotherapy (PEP) vs. active monitoring (AM) **in a 1:1:1:1 randomization, with 15 children assigned to each cell. Participants will not be permitted to receive pharmacologic or psychotherapeutic mental health intervention outside of this study protocol beginning one month prior to study participation throughout the entire 12 week trial with the exception of rescue stimulants for ADHD, as described below.** This study will explore the feasibility of conducting a combination nutrition-psychotherapy study in this population and will explore the descriptive efficacy of Ω3 along with PEP. The PBO group will receive capsules matched to the active Ω3 treatment; the AM group will attend assessments, which approximates an attention-control group. **Seven** assessments will be conducted with each child throughout a **12**-week period. **Participants receiving** PEP **will participate in** 20 to 24 **twice-weekly** therapy sessions**.** The first visit (week -1) will be used to determine study eligibility, including ability to swallow capsules, as described below. Randomization will occur at the second visit (week 0). Main outcomes (KMRS, KDRS, CGI-S/I, and C-GAS scores) will be collected for each group every 2 weeks for the first **6** weeks and every **3** weeks for weeks **6-12**. Blood draws will take place at weeks **0 and 12** to analyze fatty acid plasma levels.

Table 1 *Treatment Groups*

	Ω3 +	Ω3 – (PBO)	TOTAL
PEP +	*n*=**15**	*n*=**15**	*n*=**30**
PEP – (AM)	*n*=**15**	*n*=**15**	*n*=**30**
TOTAL	*n*=**30**	*n*=**30**	*N*=**60**

Table 2 *Study Design Summary*

	2 X 2, Supplement Double-Blind<br>Omega-3 (Ω3) vs Placebo (PBO) and<br>Psychoeducational Psychotherapy (PEP) vs. Active Monitoring (AM)			
Task	Screen	Randomize<br>Ω3+PEP, Ω3+AM, PBO+PEP, PBO+AM	**12**-week trial<br>Ongoing Assessment	Endpoint Assessment
Week	-1	0	2, 4, 6, **9**	12
Visit	1	2	3, 4, 5, 6	7

Treatments	No treatment<br>Complete pill swallowing evaluation<br>(& training, ≤ 3 sessions, as needed)	Start Ω3 or PBO<br>and PEP or AM	*n*= **15** Ω3+PEP,<br>*n*= **15** Ω3+AM,<br>*n*= **15** PBO+PEP,<br>*n* = **15** PBO+AM	
Assess-ments	P-ChIPS, ChIPS, PDD Supp & clinical eval, medical history, vital signs, PE, WASI, Grids, demo-graphics, FHS, EEAC, ATQ, SRCS, UMDQ, KDRS, KMRS, **C**-GAS	KDRS, KMRS, Grids, vital signs, **C**-GAS, CGI-S/I, DM, blood draw	KDRS, KMRS, Grids, vital signs, SE, C-GAS, CGI-S/I	KDRS, KMRS, Grids, PE, vital signs, EEAC, SE, ATQ, SRCS, UMDQ, **C**-GAS, CGI-S/I, DM, blood draw
Duration	4-5 hours	2.5-3 hours	1-1.5 hours	2.5-3 hours

Abbreviations: ATQ=Automatic Thoughts Questionnaire; **C**-GAS=Children's Global Assessment Scale; CGI-S/I=Clinical Global Impressions Severity & Improvement Scales; ChIPS=Children's Interview for Psychiatric Syndromes P-ChIPS=parent version; DM=Dietary Monitoring; FHS=Family History Screen; Grids=Medication Usage and Services Grids; **KDRS=K-SADS Depression Rating Scale; KMRS=K-SADS Mania Rating Scale; PDD Supp=K-SADS Pervasive Developmental Disorders supplement**; PE=Physical Exam+ Anthropometric measures; SE=Side Effects Review; SRCS=Self-Report of Coping Skills; UMDQ=Understanding Mood Disorders Questionnaire; WASI=Wechsler Abbreviated Scales of Intelligence

Participants

Sample. **Sixty** children aged 8-12 years will be enrolled at the rate of **2.5** per month. We have constructed an extensive referral network and are able to generate a participant pool with diverse ethnic and socioeconomic backgrounds (see Recruitment in the Human Subjects section).

Inclusion criteria: 1) aged 8-12 years (boys and girls); **2) Diagnosis of BP-NOS, using the LAMS/COBY definition described below as verified by consensus conference**; 3) full scale IQ ≥ 70; 4) child and one parent or other caregiver must be able to complete all assessment; 5) child must be able to swallow capsules (training in swallowing will be offered)**;** 6) parent and child must be willing to have blood drawn from child at visits 2 and 7.

Exclusion criteria. Participants must not have: 1) Major medical disorders (eg diabetes, epilepsy, metabolic disorder); 2) Inability to communicate in English; 3) Lack of access via phone; 4) Autism; 5) Psychosis; **6)** Active suicidal concern (e.g., "I want to kill myself", a plan for suicide, or an attempt in the past month; however, passive suicidal ideation, such as "I wish I were dead" would not exclude); 7) **three or more symptoms rated as "marked" or "severe" on the KDRS or KMRS;** 8) **Concurrent mental health intervention (pharmacotherapy and/or psychotherapy) in the past month.**

BP-NOS definition. Because criteria for BP-NOS in the DSM-IV-TR [66] are vague, researchers have created the following more specific set of minimum criteria for diagnosis [1]. Cyclothymia is not excluded from this BP-NOS definition. These criteria have been reliably used to identify youth in the Course and Outcome of Bipolar Youth (COBY) and Longitudinal Assessment of Manic Symptoms (LAMS) studies; the PIs are Site PIs for the LAMS study. The criteria are: 1) clinically significant bipolar symptoms that did not meet DSM criteria for BP1 or BP2; 2) elated mood plus 2 or more associated symptoms from DSM-IV-TR or irritable mood plus 3 or more associated symptoms; 3) a change in functioning; and minimum duration of 4 hours within a 24-hour period and ≥ 4 cumulative lifetime days meeting criteria.

Recruitment

Our previously developed referral network includes local psychiatrists, psychologists, pediatricians, family physicians, school psychologists, and other mental health professionals. The Co-PI (MAF) has generated a lengthy list of families who have requested to be called if any future studies become available. Phone contacts will be followed-up with letters and recruitment cards to be distributed to families who potentially fit study criteria. Also, Drs. Fristad and Arnold are currently following children aged 8 to 18 in LAMS (R01 MH073801, Longitudinal Assessment of Manic Symptoms). **There are approximately 20 children in LAMS who were either initially diagnosed with BP-NOS or who have converted to BP-NOS who would be in the appropriate age range for this study**. We anticipate some of these children and their families will be interested in participating **in this treatment study, as they are now quite familiar with coming to OSU for LAMS study purposes (LAMS is exclusively an assessment, not a treatment study).**

Enhancing recruitment is our recently integrated Ohio State University-Nationwide Children's Hospital (OSU-NCH) Division of Child & Adolescent Psychiatry. NCH-Behavioral Health Clinics (NCH-BH) have four community-based settings with over 100,000 outpatient visits annually **and much diversity in terms of the racial and ethnic make-up of its clientele**. Additionally, the OSU Outpatient clinic has over 6,000 visits annually. In the LAMS study, we enrolled 185 youth with elevated symptoms of mania (i.e., scored ≥ 12 on the

General Behavior Inventory Short-Form: GBI-SF, 12% of the sample were screen negatives)[67] in a 2.5-year time period (approximately 6/month); **we screened only a minority of new patients to the clinics for that study and did not recruit any existing patients, per that protocol**. The NCH-BH intake system was recently reorganized, one goal of which was to facilitate provision of information about research treatment opportunities to families prior to intake evaluations. This should further enhance recruitment. Minority recruitment will be enhanced by a four-pronged plan that includes personalized contact with and distribution of recruitment materials to key minority leaders (church leaders, physicians, community leaders). See Human Subjects for details.

Attrition

We do not know what attrition will occur in this study. This, in fact, is one purpose of the study: to determine feasibility of recruitment **and** retention for a larger, more definitive study if this pilot study suggests Ω3 and/or PEP offers clinical benefit. Of the **three** clinical trials of **DHA/EPA** Ω3 and mood in children known to the researchers, the average attrition rate **was 22**%; **three** drop-outs **(4.5% of participants)** were due to side effects **(GI disturbance)**[31,32,33,34]. This is **somewhat lower than the** 30% drop out rate in pharmacologic clinical trials of depression and bipolar disorder due to adverse events, lack of symptom improvement, or both.[13] In our large-scale RCT **of MF-PEP**, only 12% of those in immediate treatment did not complete MF-PEP. In our RCT of PEP, 20% in immediate treatment did not complete PEP. There is always the possibility in this study that participants will drop out and purchase Ω3 supplements on their own or get tired of taking them. Similarly, families may decide they are not interested in PEP therapy sessions and will terminate those early. However, both Co-PIs have experience in minimizing attrition in clinical trials of non-pharmacological treatments. In fact, Co-PI Arnold recently completed a sample of 52 in a double-blind study of 21 weeks total duration with similar visit frequency for a different supplement and experienced only **1** dropout during the double-blind phase. Frequent contact by well-trained staff and access as needed to Drs. Fristad and Arnold by study participants are among the strategies expected to minimize attrition. In our previous experience, increasing visit frequency from every 2 months to every 2 weeks in a nutritional trial decreased attrition from about 25% to <3%. Frequency of visits in this study will be every two weeks for weeks 0 to 6 and every three weeks for weeks 6 through 12. If a family decides to terminate the study early, every effort will be made to ascertain the reasons for their departure. This will be documented in the Early Termination Form (see Appendix B) administered either in a phone interview or via self-report, based on the family's willingness to share information. Differential attrition between Ω3/placebo and PEP/active monitoring and will be analyzed as an additional palatability/feasibility outcome as well as possible indicator of satisfaction with treatment results. Attrition will be used as one measure of feasibility and will be used to estimate the number of participants needed in a larger trial.

Rescue Strategies

Because the majority of children with BP-NOS also have ADHD, an exception to the medication exclusion will be that low-dose stimulants will be allowed for those whose ADHD symptoms become unacceptably impairing as stated by teacher, parent, or child or as assessed by study team. Doses will be clinically determined in consultation with co-PI LEA, who has extensive experience in use of all ADHD medications. Children will be terminated from the study for any of the following reasons: 1) they develop: a) psychotic symptoms; b) active suicidal ideation; c) active homicidal ideation; d) three or more symptoms rated as "marked" or "severe" on the KDRS or KMRS; e) dangerous behaviors that require 1:1 monitoring and/or inpatient hospitalization; f) physical diseases that require medical management incompatible with Ω3 or participation in PEP; 2) they or their parents request additional treatment (beyond rescue stimulants); 3) they become wards of the state due to actions taken by Children's Services; 4) the family moves away and is unable to complete scheduled face-to-face appointments; 5) the assessment team notes any other urgent concerns not described above that make study continuation no longer in the child and family's best interest.

Adherence

Participant adherence will be checked by standard **capsule** counts of returned unused dosage forms. Data will be recorded on a Medication Accountability Form (see Appendix C). Adherence below 86% (1 missed-dose day per week) will result in re-instruction and emphasis on the importance of taking the capsules regularly. Adherence below 70% (2 missed days/week) without good reason (such as gastroenteritis) will result in assistance with planning where to keep the doses, when to take them, and how to remember. Participants will be given pill minder boxes and capsule-administration logs to aid in adherence. All participants will be retained regardless of adherence as long as they are willing to be assessed. Differential rates of study drop-out will be examined as an outcome (see Hypothesis #2). Palatability and adherence have great practical importance both for clinical use and for the large randomized clinical trial we hope will follow this pilot study.

Ω3 Dosage and EPA:DHA Ratio

Definitive guidelines re: dosage and EPA:DHA ratio are lacking. Adult studies tend to use 1-2 g with widely varying EPA:DHA ratios. Studies of Ω3 in children vary widely in both their dose and EPA:DHA ratio (560-8075mg EPA+DHA; 7EPA:1DHA to 1EPA:4.3DHA) Thus, we determined to test a mid-range dose and a ratio that has received some past scrutiny. The Ω3 group will receive **two capsules** of 500 mg Ω3 (350 mg EPA: 50 mg DHA; 100 other Ω3) **twice** daily for a total daily dose of **2000** mg Ω3 (1400 mg EPA: 200 mg DHA; 400 other Ω3). The placebo group will receive capsules **twice** daily matched for odor and appearance with the active intervention. Ω3 and matched placebo capsules will be provided by OmegaBrite (www.omegabrite.com; see letter of support). All participants will take a multivitamin/mineral provided by the study to control for vitamin and mineral levels, **but no** other nutritional supplements **will be allowed in the four weeks preceding and throughout the 12 week study.**

<u>Concomitant Treatment</u>

Participants will be instructed not to receive other pharmacologic or psychotherapeutic interventions for mental health concerns outside of this study protocol beginning one month prior to study participation throughout the entire 12 week trial.. Rescue stimulants for comorbid ADHD will be allowed after randomization if requested by the family; this will be reported as an outcome. If potential participants have received outside treatment within the month prior to screening but are unsatisfied with this treatment, they may be randomized once they have been treatment-free for at least a month. *No participant will have satisfactory current treatment stopped or altered* to participate in this study.

<u>Procedures</u>

The Co-PIs and post-doctoral Study Coordinator will conduct initial evaluations with primary parental informants and children, and the graduate research associate (GRA) will conduct subsequent assessments. The post-doctoral Study Coordinator will provide individual PEP sessions to participants in the Ω3+PEP and PBO+PEP groups. A research pharmacist will monitor dosages and distribution of all supplement capsules. A co-investigator is a nutritionist and will review nutritional health information for general safety purposes. Visits at Weeks **0 and 12** will be scheduled in the morning to accommodate fasting blood draws. The child will be provided breakfast following the blood draw.

<u>Phone pre-screening</u>. The phone pre-screening procedure is designed to address the following questions: Does the child have a high likelihood of meeting current DSM-IV diagnostic criteria for BP-NOS? Does the child have a diagnosed metabolic disorder or other chronic health condition? Does the child attend regular classes? (Clarifying questions will be added, as needed, to determine if the child has a low IQ.) Does the child live with one or more parents/caregivers? Is it likely that the child and one or more parent(s)/caregiver(s) will be interested in participating in the research protocol (after hearing a brief description of it)? If this prescreening interview indicates possible interest in and eligibility to participate in the study, the child and parent(s)/ caregiver(s) will be invited to a screening appointment, at which time informed assent and consent will be obtained and the screen assessment completed. Parents of ineligible children will be offered referral information to seek other mental health services as appropriate.

<u>Screening</u>. Children and their parent(s)/caregiver(s) will provide written informed assent/consent and authorization to use personal health information in research, then complete screening assessment materials. The children's pill swallowing ability will be determined at this time. Children who can swallow study-sized **capsules** at screening will require no further intervention. Children unable to swallow pills will proceed with the desensitization protocol (see swallow-training protocol in Appendix D). Up to 3 visits can be scheduled, if needed, for clinical supervision of the desensitization, and/or their parents can work with them at home to complete the desensitization. Children who are able to swallow study-sized pills **and have not received treatment outside of the study for ≥ 1 month** can be scheduled for their randomization visit within a week. Children not capable of swallowing study-sized pills even after 3 training sessions will not be randomized to the study, but the number/percentage of such children initially recruited will be noted and reported as a feasibility measure.

<u>Randomization.</u> At the randomization visit, children will receive their random assignment to one of four conditions (Ω3+PEP, Ω3+AM, PBO+PEP, and PBO+AM). We will block-randomize in blocks of 4 (Ω3+PEP, Ω3+AM, PBO+PEP, and PBO+AM). While it would be desirable to balance on comorbid conditions (ie, behavior, anxiety and other disorders), this pilot sample size of **60** does not allow such a strategy. Children and their parents will be educated, based on their randomization assignment, to the appropriate regimen(s) (see Table 3 below). Children will come to the clinic for medical, nutritional and symptom evaluation every other week for the first **6** weeks and **every three weeks for weeks 6 to 12**. Children who are assigned to the PEP condition will schedule **twice-weekly** therapy sessions with the SC.

<u>Blindness of Evaluators</u>. One full-time post-doctoral study coordinator (SC) and one half-time graduate research associate (GRA) enrolled in the OSU doctoral program in clinical child psychology

will conduct interviews with parents and children at screen. To maintain the blind, the GRA will conduct baseline and follow-up evaluations as the SC will be providing therapy and thus no longer blinded to the PEP condition. While Dr. Fristad can provide general supervision of the GRA, Dr. Arnold will provide ongoing supervision of KDRS, KMRS, C-GAS and CGI-S/I ratings as Dr. Fristad will be unblinded by supervising the SC who is providing PEP. A research pharmacist not involved in assessments will dispense all Ω3 and matched placebo without un-blinding the GRA. Participants will remain blinded with placebo matched by size, color, shape, and taste.

Study time frame. Three years will be required to complete the study. The first three months will be spent hiring and training staff, preparing pharmacy packages, and initiating recruitment strategies. Enrollment will occur from month 4 to month **27**. All participants will complete all assessments by month **30**, leaving six months for data analysis and manuscript preparation (see Table 2).

Table 2 *Study Schedule*

--------------Year 1----------------*----------Year 2------*----------------------------Year 3----------------------------------

Mos. 1-3	*Mos.* ***4-27***	*Mos.* ***28****-30*	*Mos. 31-36*
Hire & train staff, operationalize study	Enroll **2.5** Ss/month, conduct assessments and treatment	Complete all treatment and assessments	Analyze data, prepare manuscripts & submit R01

Study coordination. Comprehensive research team meetings will be held weekly initially, then at least **every other week.** These meetings will provide a forum for the entire multidisciplinary team to discuss methodological issues related to assessment, intervention, data management and analysis. **No information that could un-blind the GRA will be discussed while the GRA is present.**

Instruments. **See Appendix E for descriptions of each instrument used in this research plan.**

Consensus diagnosis and ongoing ratings. The assessment team (Drs. Fristad and/or Arnold, the post-doctoral Study Coordinator and the psychology graduate research associate [GRA]) will meet within **2 business days** of screening assessments prior to randomization. During these meetings, study eligibility decisions will be finalized and consensus diagnoses will be determined for participants. Consensus diagnoses will utilize all information obtained. Data from ChIPS/ P-ChIPS will **follow the "either-or" rule** (ie, counting a symptom as positive if either the child or parent endorses it) unless there are clear and compelling reasons to disregard information (eg, if the child responded "yes" to everything asked; if the parent/caregiver exaggerated **all answers**).

Nutritional Assessments

These will be conducted at Weeks **0 and 12** at the OSU Clinical Research Center (CRC) (a 5-minute walk from the OSU Childhood Mood Disorders Lab). Blood draws will be completed by research nurses trained in pediatric blood draws and nutrition assessments by research dieticians highly experienced in these procedures (see letter of support). Dr. Belury will direct the fatty acid assays.

Assays of fatty acid levels. To determine the extent that participants consumed and accumulated Ω3 fatty acids, we will analyze non-esterified fatty acids by gas chromatography of plasma levels of EPA and DHA. As we have shown[46], plasma fatty acid composition reflects fatty acid intake of the past few days to week of supplementation with Ω3 supplements. Therefore, in addition to participant-reported adherence, plasma fatty acid analysis should reflect actual intake of supplements over several days prior to each blood test. In a recent pilot study (as part of the NCI-NIH HER-2/neu and dietary fat: gene-nutrient interactions in breast cancer grant for which Dr. Belury is Co-Investigator), we found a significant accumulation of EPA and DHA in plasma within one month in adults receiving approximately equivalent doses (expressed as Ω3/kg body weight) compared to doses proposed here for children. Levels remain elevated in plasma for up to 6 months if participants continue consuming supplements. Our preliminary findings in adults demonstrate using the dose proposed in the present study will likely result in an approximate two-fold increase of EPA and DHA levels.

Fatty acid composition will be analyzed using a two-step procedure described previously.[68] Total lipids will be extracted using the method of Bligh and Dyer.[69] Fatty acid methyl esters (FAMEs) will be prepared from each lipid fraction by incubating samples with tetramethylguanidine at 95°C[70] and quantified using a Hewlett Packard 5890 gas chromatograph equipped with an auto-sampler ChemStation software (Agilent Technologies, Meriden, CT), FAME ionization detector and a 30-m Omegawax 320 capillary column (Supelco Co.).[46] Helium flow rate will be 30 ml/min and oven temperature will be programmed to start at 175°C then ramped to 220°C at 3°C per minute. Identification of FAMEs will be accomplished by comparing retention times of samples to retention times of authentic standards (Supelco Co.). The internal standard heptadecanoate (17:0) will also be used to quantify absolute amount of fatty acids in ng/ml. Dr. Belury (Co-Investigator) will direct these analyses. Besides these specialized assays, routine cholesterol fractions and triglycerides will be checked.

Dietary monitoring. Diet will be monitored with the standardized USDA Automated Multiple Pass Method[71] in addition to Food Frequency Questionnaires (FFQs). Using a 5-step multiple-pass method, children

and parents will be interviewed about the child's food/beverage consumption for the previous 24-hour period.[72,73]The 24 Hour Recall or Typical Diet Form (see Appendix F), supplemented by samples of plastic and paper food models and portion estimates, will be used to increase precision in the data gathered. This method reduces underreporting of intake due to repeated opportunities to recall foods and beverages.[71]

Dietary records will be entered into the Nutrition Data System for Research (NDS-R) software by a research dietician [74,75] This nutrient analysis program is maintained by the Nutrition Coordinating Center (NCC) at the University of Minnesota's School of Public Health. The NCC maintains a database containing 139 nutrients, nutrient ratios, and food components. The NDS-R software provides data for 18,000 foods and 8,000 brand names of foods. The NCC database is derived from the USDA database, scientific literature, food manufacturers and foreign food composition tables. Individual and group data and comparisons to national recommendations can be exported into spreadsheet or database analysis programs for further statistical comparisons. Food groupings can be reviewed and scored according to the method developed by Kant[76] using the data generated by NDS-R to determine if diet pattern has changed over the study. Likewise, the diversity score can be determined by the number of food groups consumed daily[77] and compared to food groups recommended by the Dietary Guidelines for Americans.[74,75,78,79]

Interviewers

Non-nutrition assessments will be conducted by project personnel hired for this study. One post-doctoral study coordinator (SC) and one half-time graduate research associate (GRA) enrolled in the OSU doctoral program in clinical child psychology will conduct interviews with parents and children respectively at screen. To maintain the blind, the GRA will conduct follow-up evaluations as the SC will be providing therapy and thus no longer blinded to the therapy treatment condition. While Dr. Fristad can provide general supervision of the GRA, Dr. Arnold will provide ongoing supervision of KDRS, KMRS, C-GAS and CGI-S/I ratings as Dr. Fristad will be supervising the SC who is providing PEP, and thus, will not be blind to psychotherapy assignment. Ideally, the same staff will remain on the study for its duration, allowing for continuity of assessors. An interviewer can complete four interviews, on average, per week, including all requisite form completion, given the standard rate of no-shows and cancellations. Thus, interviewing staff will also be able to oversee various project tasks to maximize their time management. Interviewers will go through a detailed training program led by the Co-PI (MAF) prior to conducting interviews. The training program will include extensive instructions on child psychiatric syndromes; how to administer questionnaires; information regarding clinical and ethical issues involved in interviewing; practicing, observing and rating interviews; performing mock interviews and receiving feedback from experienced study personnel; and undergoing reliability checks. Project personnel will observe and concurrently rate mock interviews. When new interviewers have achieved reliability on all administered interviews (*kappas* $\geq$ .70) they will be videotaped conducting live interviews. Videotaped interviews will be reviewed by Drs. Fristad and Arnold. After reliability is achieved in live interviews (*kappas* $\geq$.70), project personnel can interview independently. All families will be asked to consent to being videotaped. Ten percent of tapes will be randomly chosen to be reviewed and independently rated. If these checks uncover rater "drift", that interviewer will be required to repeat training procedures until the reliability standard (*kappas* $\geq$ .70) is regained. Approximately one hour per week of on-site supervision will be provided on a weekly basis. Our Multi-family Psychoeducation Group (MFPG) study experience has taught us that kappas are actually above .70 once proper training is completed, and ongoing supervision appears to successfully assist in maintaining high levels of reliability (range for ChIPS, P-ChIPS and mood rating scales: *kappa* = .70-.85).

Therapist

The SC will conduct all PEP treatment with families enrolled in the study. S/he will have completed doctoral training and an internship in clinical child psychology, be trained by the Co-PI (MAF) in conducting Psychoeducational Psychotherapy (PEP) and receive weekly individual supervision from the Co-PI to ensure treatment adherence and address any questions or concerns raised by participating families or the therapist.

Participant Reimbursement

In recognition of travel costs, other expenses, time (possibly lost time from work), and inconvenience, the child and parent will be paid upon completion of each assessment. **See payment schedule in Human Subjects Section.**

Potential Problems/Limitations and Alternative Strategies Considered

We considered studying only one intervention in this trial; however, TADS [37] provides compelling rationale for studying combination treatment **in adolescent depression and a series of other studies have indicated superiority of combined treatment for other disorders (PDD, anxiety, ADHD)**. Additionally, the 2X2 design allows us to examine the potential efficacy of each intervention separately, as well as together.

Data Analysis

The data analytic plan has been developed in conjunction with Dr. William Gardner, a developmental-quantitative psychologist. Dr. Gardner will plan and supervise analyses in collaboration with the Research Team. It is recognized in this pilot study that we will focus primarily on determining feasibility and potential effect sizes. Only secondarily will we conduct exploratory examinations of the data.

Sample size determination. Due to the exploratory nature of this study, the sample size of **60** was chosen purely for practical reasons of recruitment, time and monetary limitations of an R34 grant. The 2X2 design (with 15 participants per each treatment combination) grants more power to examine potential moderators of treatment response to Ω3 and to PEP. This proposed pilot study should provide necessary information for planning a large-scale, definitive study. Negative findings in the pilot study might be attributable to insufficient power to detect meaningful effects with high confidence; in a sufficiently powered study, a negative finding is more conclusive. While the proposed pilot study is not being used to determine an effect size for a subsequent large scale study, this pilot study will provide a check to see if the desired effect size ($d \geq 0.5$) is plausible.

Operationalized variables. Primary outcome measures are the KMRS and KDRS. . Additional outcome measures include **C**-GAS, CGI-S & CGI-I. Potential mediators include: *Adherence*--% of pills consumed, EPA and DHA levels, and % of PEP sessions attended; *Negative Automatic Thoughts*—ATQ; *Coping Strategies--SRCS; Knowledge of Mood Disorders*—UMDQ; *Family climate*—EEAC. Potential moderators include: *baseline EPA and DHA levels, family history of mental illness-* Family History Screen; *Children's Axis I Comorbid Disorders:* Consensus diagnoses of behavioral, anxiety and 'other" disorders, as derived from the ChIPS or P-ChIPS **and PDD Supplement (while autism is excluded, children with PDD-NOS or Aspergers might be included in the study);** *Psychosocial stressors*—as obtained on the ChIPS and P-ChIPS. **Treatment history** and subsequent changes in **treatment (ie, use of rescue stimulants)** will be quantified using the Medication and Service Providers Grids. Side-effects will be monitored using the Side Effects Review.

Exploratory data analysis and missing data. Exploratory data techniques will be used to examine all distributions for outlying or aberrant observations.

Intent to treat (ITT). All participants will be analyzed as members of the group to which they were randomized, regardless of actual adherence with the treatment regimen (as defined by pill counts and number of PEP sessions attended).

Model. A linear mixed effects model (LME)[80] will be fit to each outcome variable. The random effects are intercept and slope; which posit that each participant has a "personalized" linear response to treatment. The fixed effects are treatment X time interactions, which measure systematic differences in the rate of change. The assumption of a common initial mean for all participants is justified by the randomization of participants into treatment groups. Here an additional term for random "noise" allows for measurement/replication error. A major advantage of the LME model is that it does not require a balanced design; in particular, participant effects can be estimated using incomplete data. Thus there is no need for imputation as long as missing data are missing at random.[81]

Table 3. *Summary Table—Treatment Groups*

1. PBO+AM	2. PBO+PEP	3. Ω3+AM	4. Ω3+PEP

Major hypothesis testing.

1. *Feasibility--Participants can be recruited in* ***a two–year period****.* Descriptive statistics will be used to characterize the recruited sample **and time frame. All 4 groups (1-4)**
2. *Attrition in PBO+AM vs other 3 groups at Week* ***12***: We will use negative binomial regressions[73] to analyze these data. **Group** 1 vs 2/3/4
3. *Ω3 will have a clinically meaningful effect size on* ***depressive and manic*** *symptoms reported on the* ***KDRS and KMRS, respectively****, compared to placebo at Week* ***12***: The effect size of interest is the differential rate of change attributable to Ω3 versus placebo, expressed as relative to the (assumed) common standard deviation of individual rates within group. Restricted maximum likelihood estimation (REML) will be used to reduce bias when estimating the between and within group variance components of the random slopes (rates of change). The effect size is estimated as $d = \left(\bar{b}_t - \bar{b}_c\right) / s_b$, where $\bar{b}_t$ and $\bar{b}_c$ are the mean rates of change in the treatment and control groups respectively, and s_b is the pooled within group standard deviation of the individual slopes. If $d \geq .5$, that will be considered *prime facie* evidence for proposing a large-scale investigation. **(However, the effect size found will not drive the power analysis for reasons elucidated by Kraemer).** As a secondary means of assessing change, chi square analyses will be used to determine the relationship between response (as defined above) and treatment status (PBO vs Ω3). 1/2 vs 3/4

4. *PEP will have a clinically meaningful effect size* ***(d ≥.5)*** *on* ***depressive and manic*** *symptoms reported on the* ***KDRS and KMRS, respectively****,, compared to active monitoring at Week 12*: this will be analyzed in the same manner as #2 above (PEP vs. AM). 1/3 vs 2/4
5. *Combined treatment (Ω3+PEP) will demonstrate a clinically meaningful additive effect (d≥.25) on depressive* ***and manic*** *symptoms reported on the* ***KDRS and KMRS, respectively****, compared to either individual treatment (Ω3, PEP) at Week* **12**. Comparisons will be made as described above. 4 vs 2, 3

Exploratory analyses.

1. To explore rates of change and timing of clinical response we will graph clinical responses to Ω3 and PEP on the **KDRS, KMRS** and CGI-S/I to see if and how soon any benefit is noted and when it plateaus. Main outcome variables will be plotted in ITT graphs where the horizontal (ie, time) axis spans the entire **12** week duration of the study. We will examine whether improvement is sustained (constant rate of change), plateaus or attenuates (decelerating improvement) as the study progresses. Ω3, 3/4; PEP, 2/4
2. To examine clinical significance more broadly, hypotheses #3-5 will be examined via graphs of a variety of outcome measures, including global functioning (C-GAS). Measures that appear particularly sensitive to change will be considered for any larger subsequent study; others will be dropped to minimize participant burden. Same as Hypotheses 3-5 above
3. To explore possible mediating effects, we will examine graphs of the associations between rate of change in clinical outcome and changes in EPA and DHA blood levels, number of PEP sessions attended, and changes in negative automatic thoughts (ATQ), coping strategies (SRCS), knowledge of mood disorders (UMDQ), and family climate (EEAC). Ω3, 3/4; PEP, 2/4
4. To explore possible moderators of outcome, we will examine graphs of the associations between rate of change in clinical outcome and baseline lipid blood levels, comorbid disorders (ascertained on ChIPS and P-ChIPS), psychosocial stressors (ascertained on ChIPS and P-ChIPS), family history of mental disorders (FHS), and treatment history (Service and Med grids). 1-4
5. To track adherence over time, parent- and child self-reported rates of capsule ingestion will be tabulated against staff **capsule** counts. Then both self-reported rates of capsule ingestion and **capsule** counts will be correlated with EPA and DHA blood levels after 12 weeks to check validity of the former. 3-4
6. Changes in EPA and DHA blood levels will correlate with changes in **mood** ratings (as measured by the **KDRS+KMRS**) at $r \geq 0.5$.
7. Determine rates of **previous** treatment utilization and side-effects for all participants (as measured by the Medication and Services Grids and Side Effects Review, respectively).
8 **Results from the analyses described above will be compared and contrasted with those found in a parallel study of children with depression.**
