# Supplementary material for: Body weight affects ω-3 polyunsaturated fatty acid (PUFA) accumulation in youth following supplementation in post-hoc analyses of a randomized controlled trial
Source: PLoS One. 2017 Apr 5;12(4):e0173087. doi: 10.1371/journal.pone.0173087 (PMC5381773; doi:10.1371/journal.pone.0173087)
Supplement: S2 Trial protocol — (PDF) [file pone.0173087.s003.pdf]

RESEARCH STRATEGY

Significance

Childhood-onset depression (major depressive disorder [MDD] and dysthymic disorder [DD]) is a major health concern because of immediate consequences for children's functioning at home, at school, and with peers and subsequent risks for recurrence of depression or other mental disorders.[1] Research suggests 20% of youth experience MDD before age 18.[1] Children with depression are at greater risk for developing disruptive behavior and substance abuse disorders.[1, 2] Given the less than robust response to **medication**, alternative physiologic interventions have been considered, **such as** omega-3 fatty acids (Ω3).

Pharmacologic Findings. The US Food and Drug Administration[3] meta-analysis of antidepressant treatment in children and adolescents found a 2-fold increase (4% vs. 2%) in risk for suicidal behavior or ideation for medication over placebo, **and** mandated a black box warning for all antidepressants for youth. In addition, efficacy of antidepressants for children **is** in question. **Of** 15 trials for MDD, only 9 included children **age 12 and under** (539 children out of 2910 total participants,18.5%) and none focused solely on children[4]. Only fluoxetine has been shown to be significantly better than placebo in children; **possibly** due to the high rate of placebo response. No combination trials of medication and psychotherapy have been conducted with children; the Treatment of Adolescent Depression Study (TADS) demonstrated the superiority of combined treatment for adolescents with major depression.[5, 6] Given concerns about treatment-induced suicidal ideation and the questionable effectiveness of antidepressants in children, parents may seek other treatment options, such as psychotherapy or dietary supplements.

Psychotherapy Findings. Despite the prevalence of and impairment experienced by depressed children, RCTs of psychotherapy with **children diagnosed with depressive spectrum disorders are lacking**.[7] A recent review of evidence-based psychosocial treatments for children with depression **showed** cognitive-behavioral therapy (CBT), in child-only groups and child group plus parent components, was the only "well-established" intervention approach[7], **but these were for children with "elevated depressive symptoms" or "at risk" for depression, rather than children diagnosed with depressive spectrum disorders.**

Omega-3 Fatty Acids (Ω3) and Mental Health. Epidemiological studies comparing rates of mood disorders in countries with varied rates of fish consumption **found significant negative correlations (*r* = -.84, *p*<.005[8] & *r* = -.74, *p*<.0001[9]). DHA supplementation in boys has been found to increase activity in attention networks in the prefrontal cortex during sustained attention tasks.[10]** Owen **et al**[11] posited four pathways for Ω3 depletion that may be linked to depression. First, ratios of DHA to other fatty acids affect membrane fluidity and **enzyme** functioning, ion channels, and receptor binding. Second, Ω3 concentrations impact neuroplasticity and cell survival. Third, Ω3 concentrations affect gene expression. Fourth, Ω3 availability **suppresses** production of proinflammatory cytokines, which are elevated in depressed patients and inhibited by some antidepressants.[11] High resolution structural MRIs **show** people with higher intake levels of Ω3 have greater grey matter volume in the anterior cingulate cortex, the right hippocampus, and the right amygdala. These brain areas **are** involved in emotional arousal and emotion regulation **and are** reduced in people with mood disorders.[12] **Others** have demonstrated lowered T_2 values, suggestive of increased membrane fluidity, in patients with bipolar disorder who take Ω3.[13]

Ω3 may improve symptoms in a variety of psychiatric disorders including mood disorders (see Appendix A for summary of studies). **Pediatric studies show benefits for hyperactivity and stereotypy in autism,[14] improvements in inattention, hyperactivity, oppositional/defiant behavior, and conduct problems in children with ADHD,[15,16], and improvements in reading, spelling, and behavior in children with developmental coordination disorder.[17] A recent 12-week RCT of 81 individuals, age 13-25, with "ultra-high risk" of psychotic disorder was followed by a 40-week monitoring period.[18] Two of 41 participants in the Ω3 group (4.9%) and 11 of 40 in the placebo group (27.5%) developed psychotic disorder. Compared to the placebo group, those in the Ω3 group showed significantly lower levels of positive symptoms (*p* = 0.01), negative symptoms (p = 0.02), and general symptoms (*p* = 0.01) and displayed improved functioning (p = 0.002).[18]** In addition to **safety**, Ω3 may actually provide multiple health benefits.[19, 20] **Besides** decreasing psychiatric symptoms,[21, 22] Ω3 have been shown to significantly improve cardiovascular and metabolic health and decrease body fat, both independently and in combination with regular exercise.[23] **The latter** is particularly important, as **many** current treatments for mood stabilization are associated with significant weight gain, obesity and metabolic disorders.

Adult mood disorder studies. **Studies conducted to date on Ω3 in the treatment of mood disorders in adults are summarized in Appendix A.** Although available studies are heterogeneous **in methodology and results, a meta-analysis showed** a significant antidepressant effect of Ω3 in the overall sample (*N*=329,

ES=0.61, *p*=.003), in patients with clearly defined depression (*n*=222, *ES*=0.69, *p*=.002) and bipolar disorder (*n*=105, *ES*=0.69, *p* =.0009).[24]

*Child **mood** studies.* **One RCT in depressed children and one RCT and two open-label trials in youth with bipolar spectrum disorders have been reported. A 2006 RCT** examined Ω3 in 28 children aged 6 to 12 with MDD.[25] Participants were randomized to Ω3 (2:1 EPA: DHA ratio, 380-400 mg EPA and 180-200 DHA) or placebo **for 16 weeks**. Eight children dropped out: 5 for noncompliance, 1 for a manic episode, 1 for an endocrine workup, and 1 for nonresponse. No clinically significant side effects were reported. Results **in the 20 completers** demonstrated highly significant effects of Ω3 on depressive symptoms. Seven of ten children who received Ω3 had >50% reduction in depressive symptoms compared to no placebo-treated children. Four of ten children receiving Ω3 met remission criteria; **none of 10 in the placebo group did.** Most of the treatment response noted at week 16 had been achieved by week 12. The lack of clinically relevant side effects reported by participants suggests Ω3 may have a better risk: benefit ratio than traditional anti-depressants.[25]

A 6-week open trial of 360 mg EPA and 1560mg DHA daily in 18 youth with BP1, BP2 or BP-NOS found Ω3 red blood cell levels and levels of EPA and DHA increased significantly from pre- to post-supplementation. Clinician ratings of depression and mania decreased significantly (*p* =.002; *p* =.004) and global functioning increased significantly (*p*<.001). Parent-rated internalizing and externalizing behaviors decreased significantly (*p* =.009; *p* =.014).[26] Another research group conducted an 8-week open trial of Ω3 (7:1 EPA:DHA ratio) as monotherapy (with stimulants allowed) for 20 outpatients aged 6-17 with bipolar disorder.[27] Participants received 1.3 to 4.3 g of Ω3 daily with most (85%) taking ≥2.0 g/day. **Four dropped out for** lack of efficacy, not adverse events. Ω3 caused few mild side effects, mostly gastrointestinal. By Week 8, mania symptoms decreased by 30% in 50% of participants and decreased by 50% in 35% of participants. The 17 who took ≥2.0g of Ω3 fatty acids per day showed greater improvements than those who took <2.0 g per day. Depression ratings also decreased significantly **showing a 10.4 ± 9.9 point reduction on the Children's Depression Rating Scale (CDRS; *p*=0.002)**. By study end, 40% of participants were rated much or very much improved for depression and mania. The authors suggest dosing studies would benefit this body of research, especially in pediatric samples.[27]

Lastly, a RCT of flax-seed oil (alpha-linolenic acid [ALA]) vs. olive oil in 44 youth aged 6-17 with BP1 or BP2 found groups did not differ on clinician rated depression and mania symptoms or global functioning. Gracious hypothesized low conversion from alpha-linolenic acid to EPA and DHA, which emphasizes the importance of studying EPA and DHA directly.[28]

<u>Combination treatment</u>. Studies have indicated combination treatment is advantageous in treating anxiety (Child-Adolescent Anxiety Multimodal Study [CAMS][29]), adolescent depression (TADS[5, 6]), pervasive developmental disorders[30], and ADHD (Multimodal Treatment Study of Children with ADHD [MTA][31], [32], [33], [34]). Combination treatment has not been examined in children with depression.

Innovation

Practice parameters for treatment of depression in youth developed by the American Academy of Child and Adolescent Psychiatry (AACAP) include "psychoeducation, supportive management, and family and school involvement" (p. 1510).[1] The current study will examine efficacy of an intervention **combining** the AACAP-recommended elements of treatment with the CBT techniques proven helpful to groups in school-based interventions. Individual Family Psychoeducational Psychotherapy (IF-PEP) is a manualized treatment based on Multi-Family Psychoeducational Psychotherapy (MF-PEP), an empirically-supported group intervention based on psychoeducation, cognitive-behavioral, and systems theories.

Psychoeducational psychotherapy (PEP). Within a psychoeducational approach, psychiatric disorders are presented as "no-fault" **(i.e., no-blame)** illnesses that families can learn to control and manage. The importance of separating symptoms from the individual is emphasized.[35] Family members are encouraged to "back off" to help the patient recuperate from the current episode. Simultaneously, the family is offered support, validation, and recognition for their own difficult experiences in living with **the child's mood disorder.** Family members are taught that patients are particularly vulnerable to stress and tension; thus, therapists work with families to reduce the level of stress and tension in their homes. Family members are taught to set limits in a matter-of-fact rather than a critical or hostile way.[36] **Improvement of** communication, problem solving and coping strategies can lead to restoration of hope for recovery and decrease family dysfunction. **Family members are supported** in getting their own treatment if necessary (the genetic component of depression means other family members may also experience depression). **Goals include** strengthening the parent-child bond and helping children and parents feel competent to fight depression now and **in** future recurrences.

Research findings. Few studies have examined psychoeducation with families of impaired youth, despite clear evidence that **they** have many unmet needs and that assisting families to shift from "emotion fo-

cused coping" to "problem focused coping" is useful (Sloper, 1999).[37] Several authors **describe** psychoeducation programs developed for parents of psychiatrically hospitalized children,[38] outpatient youth and their parents,[39] or parents of outpatient youth with schizophrenia;[40] these articles provide limited family outcome data.[41] **In one study,** after a two-hour presentation about depression **to families of mood-disordered adolescents,** parents significantly increased their knowledge and modified some dysfunctional beliefs about depression.[42] **Another** study demonstrated 34 youth aged 5 to 17 diagnosed with BPD who received specialty clinic medication management plus psychoeducational intervention experienced significant decreases in bipolar symptom severity and increased global functioning.[43] **An open** pilot of an adolescent version of Family Focused Treatment (FFT), which includes psychoeducation and skill-building, with 20 adolescents with BPD and their families **found** decreased ratings of depression and mania as well as fewer behavior problems.[44] **Only** one investigator (Co-PI MAF, results described below) has conducted randomized clinical trials with depressed children.

<u>**Summary.**</u> **Childhood depressive disorders are associated with lifetime impairment. Evidence-based treatments for childhood depression are limited and medications, in particular, carry with them a number of serious concerns, including suicidal ideation and behaviors. Previous studies suggest both Ω3 and IF-PEP will be beneficial in this population.[25, 26, 27, 45, 46] Studies of Ω3 are promising as safe and potentially beneficial in the treatment of depression, but much more research is needed to determine their overall benefit. Our research team has developed, refined, and tested various forms of psychoeducational psychotherapy for mood-disordered children. Both MF-PEP and IF-PEP are well received by parents and children. While MF-PEP offers the unique advantage of social support from adult and child peers, it will probably not be feasible to offer except in larger clinic settings, thereby depriving a large number of children and families from accessing this specialized care. IF-PEP, on the other hand, is more readily provided. A commercially available treatment manual will also be available for IF-PEP within the year; thus, if this trial is effective, dissemination will be aided by a readily accessible treatment manual and training materials. A more rigorous evaluation of IF-PEP's efficacy and exploration of variables that mediate and moderate treatment outcome is warranted. This application would be the first combination treatment study of Ω3 and psychotherapy in children with depression aged 8 to 12. Results will indicate the feasibility and advisability of further empirical investigation by obtaining an estimate of the impact of Ω3,IF-PEP and their combination on ratings of depression and global functioning. This study will explore two interventions free of the side effects of antidepressants, and may lead to treatment with a more favorable risk-benefit ratio for children with depressive disorders.**

<u>**Approach**</u>

We have assembled a team with specific expertise in the areas relevant to the application. <u>Dr. Fristad</u> has successfully recruited children (ahead of schedule and over proposed census) in the age range of the proposed study who are diagnosed with mood disorders for studies of adjunctive psychosocial interventions or phenomenologic studies. She has retained the majority of these children and their families up to 52-months and for 10 to 20 visits, depending on the study. **She also has experience in her clinical practice using omega3 with depressed youth.** <u>Dr. Arnold</u> has successfully conducted trials of nutritional interventions for school-aged children diagnosed with ADHD **(including a study of essential fatty acids)** with notable retention rates over Phase 1 and Phase 2 trials **and has experience with clinical use of Ω3**. <u>Dr. Belury</u> is a human nutritionist internationally known for her research with PUFA to influence various medical conditions.[47, 48] She is currently working as a Co-Principal Investigator with Jan Kiecolt-Glaser, PhD, another OSU investigator, on a study of omega-3, inflammation and stress in adults. In the current application, Dr. Belury will oversee examination of dietary fatty acids. <u>Mr. Votolato</u> is a highly experienced clinical research pharmacist with extensive collaborative experience in running adult and child mood disorder clinical trials. <u>Dr. Gardner</u> is a developmental-quantitative psychologist who has contributed significantly to the mental health literature with focused work on the treatment of youth depression. He has an active NIMH grant examining suicidality among youth prescribed His training includes a master's degree in statistics and doctorate in psychology.

<u>*Preliminary Studies*</u>

Over the past 17 years, our research group has pursued efforts to develop and empirically validate MF-PEP and IF-PEP as effective treatment modalities for families of children with mood disorders. We have: a) identified developmental adaptations required to modify adult-based programs for use with families of mood disordered children or adolescents;[49] b) developed specific therapeutic techniques to work with young children;[35,50] c) developed treatment manuals for MF-PEP and IF-PEP;[70, 51] d) developed and tested instruments to assess variables of interest to our work;[52, 53, 54, 55, 56] e) implemented initial outcome studies;**[57, 58]** f) implemented three RCTs–pilot studies (*N*=35) for MF-PEP [51, 59, 60,] and IF-PEP (*N*=20)[45] and a large scale (*N*=165)

RCT of MF-PEP (R01 MH61512);[61, 62, 63] **g) trialed the 24-session version of IF-PEP in two children with bipolar disorder[46]** and h) published a treatment manual.[70] We discuss our three RCTs in further detail below.

PEP includes psychoeducation, support, and skills development. Parents and children are given their own workbook and families receive project handouts at the end of each session. Sessions begin with a brief "check-in" meeting of parents and children, during which the previous week's projects are discussed.

Small Scale MF-PEP RCT: The Ohio Department of Mental Health (ODMH) Study. We studied 35 children, aged 8-11, with major mood disorders and 47 of their parents (14 fathers, 33 mothers).[51, 59] Primary mood diagnoses included: MDD (n=13, 37%); dysthymic disorder (n=6, 17%); bipolar I (*n*=5, 14%); and bipolar II (*n*=11, 31%). All families were encouraged to continue treatment-as-usual (TAU) during the study. Eighteen families were enrolled immediately into a six-session MF-PEP (IMM+TAU) condition; 17 families were enrolled into a 6 month waitlist condition (WLC+TAU), after which they received MF-PEP. Four assessments were conducted: Time 1 (T1) at baseline; T2, 2 months after study enrollment (post-MF-PEP for IMM); T3, 6 months after study enrollment (pre-MF-PEP for WLC); and, T4, for the WLC only, post-MF-PEP.

IMM parents demonstrated: 1) significantly more knowledge than the WLC families immediately after MF-PEP, sustained at 6-month follow-up [Group X Time (1-3): $F(1,28)=7.85$, $p<.009$]; and 2) a gain in positive family interactions [Group X Time (1-3): F (1, 26) = 4.09, p < .05]. Pre-post intervention gains in positive ratings were similar for the IMM and WLC groups [MF-PEP T1-T2 scores vs WLC T3-T4 scores: $t(28) = 0.76$, *ns*]. By T3, 82% of IMM families reported an improved ability to obtain appropriate services, compared to 20% of WLC families ($X^2=7.43$, $df=1$, $p<.01$). Immediately following treatment, parents reported multiple benefits, including: 73%, increased knowledge about mood disorders; 54%, receipt of social support; 31%, attitude changes; and 31%, coping skills acquisition. By 4 months post-treatment, 71% of parents reported a positive attitudinal shift. IMM children reported a significant gain in perceived social support from their parents from T1 to T3, compared to WLC children [Group X Time: $F(1,26)=10.77$, $p<.003$]. Similarly, IMM children reported increased perceived social support from peers compared to WLC children from T1 to T3 [$F(1,24)=2.55$, $p<.12$]. **Increases in positive family interactions were comparable** for children with depressive spectrum **vs** bipolar spectrum disorders. Parents of children with depressive spectrum disorders showed larger increases in knowledge about mood disorders compared to parents of children with bipolar spectrum disorders. Parents of depressed children had significantly lower knowledge scores than parents of children with bipolar spectrum disorders at baseline; scores from the two groups were comparable post-treatment.[60]

Large Scale MF-PEP RCT: The National Institute of Mental Health (NIMH) Study (R01-MH61512). We conducted an efficacy trial of MF-PEP in 165 children aged 8-11 with mood disorders and their primary and secondary caretakers.[61, 62, 63] All families were encouraged to continue with TAU **throughout the 18-month study**. Seventy-eight families were randomized into immediate treatment (IMM=MF-PEP+TAU) and 87 families into a 1 year wait-list condition (WLC= WLC + TAU). After 1 year, WLC families participated in MF-PEP. Assessments occurred at baseline (Time 1, T1), 6 months (T2), 12 months (T3), and 18 months (T4). Participants were predominantly male (73%) and White (91%); average age at study entry was 9.9 years (SD=1.3).[62] Most came from two-parent (including step-parent) families (74%). Incomes were equally distributed. Nearly all of the participants had comorbid behavioral disorders or anxiety disorders. Study children had, on average, experienced a long period of impairment (MDD, M=65.6 weeks, SD=99.2; and DD, M=87.2 weeks, SD=115.8).

MF-PEP+TAU was associated with lower Mood Severity Index (MSI). MSI equalized potential contributions of manic and depressive symptoms as ascertained via the Children's Depression Rating Scale (CDRS-R)[64] and Young Mania Rating Scale (YMRS)[65] using the following equation: (CDRS-R score -17 * [11/17])+ MRS score). Scores lower than 10 represent minimal symptoms, scores 11-20 reflect mild symptoms, 21-35 reflect moderate symptoms and over 35 indicates severe symptoms**.[62]** **T3** MSI scores **decreased significantly more for the IMM than the WLC group in ITT analyses** ($\chi^2 = 4.55$, df=1, p = 0.0329). **Findings** were more pronounced for the Time 3 completer sample ($\chi^2=6.98$, df=1, p=0.0083). MSI **scores from** Baseline to 12-month follow up for the IMM + TAU group **indicate** a decrease from the high **to low** end of moderate symptoms. The WLC+TAU group remained near the upper end of moderate symptoms until they received **MF-PEP** between 12- and 18-month follow-up, at which point they functioned in the mild-moderate range. By 18-month follow-up, the IMM+TAU group scored in the mild range of symptom severity. Pre-post treatment decrement in MSI scores was similar for the IMM and WLC groups (3.24 and 3.50 units per six months, respectively).

Small Scale IF-PEP RCT: The Ohio Department of Mental Health (ODMH) Study. Transportability issues regarding MF-PEP led us to develop a second form of intervention, IF-PEP. These issues are as follows: First, many community-based clinicians, especially those in private practice, do not work in settings conducive to leading groups, as their case load at any given time would not support running a group for children of similar ages with the same diagnosis. Second, some families may not feel comfortable sharing personal information in

a group setting (eg, if a parent holds a more public position, such as a physician or teacher in the community, two examples that occurred during our MF-PEP recruitment) and may prefer more privacy to work on their clinical issues. Third, if clinics offer MF-PEP only once or twice a year, a family with a newly diagnosed child may not wish to wait that long for immediate, tailor-made assistance. In this case, the family could participate in IF-PEP then potentially participate again in MF-PEP to obtain the additional benefit of social support from meeting other parents and children in similar circumstances. Finally, practical issues of how to bill for multiple therapists providing simultaneous service can be a barrier to treatment implementation.

We conducted a pilot study examining the efficacy of IF-PEP in 20 children with BPD and their parents.[45] Methodology was very similar to the MF-PEP NIMH study, although the assessment was less extensive and data were collected only from the child and a primary parental informant (all mothers). Ten families were randomized to immediate IF-PEP+TAU (IMM); 10 families to a 12-month WLC+TAU (WLC). After one-year, WLC families participated in IF-PEP. Four assessments occurred over 18-months: baseline (Time 1, T1), 6 months (T2), 12 months (T3), and 18 months (T4). IF-PEP session content and projects were nearly identical to that of MF-PEP. However, IF-PEP treatment was delivered in sixteen 50-minute sessions, alternating between parent-only sessions and, after the requisite "check-in," child-only sessions. Fifteen sessions dealt with specific issues associated with BPD. One "in the bank" session was available for use at anytime for families to deal with crises and/or review previous material. The MF-PEP "group games" treatment component was deleted, but a unit on "Healthy Habits" was added that focused on improving sleep hygiene, diet and exercise. This was done because: impaired sleep can trigger a manic episode;[66] exercise can decrease depression;[67] and many medications used to treat BPD are associated with significant weight gain, with nutritional and behavioral alterations needed to combat this adverse side effect.[68]

Most **child participants** were male (85%), White (90%) and came from two-parent (including step-parent) families (65%). Incomes were broadly distributed. Primary mood disorder diagnoses include: Bipolar I Disorder (40%--10%, manic; 30%, mixed); Bipolar II Disorder (35%); Bipolar Disorder—Not Otherwise Specified (BP-NOS: 25%). Children had, on average, been impaired a considerable length of time (in weeks, manic episode, *M*=68.9 *SD*=125.7; MDD, *M*=73.5, *SD*=121.8; and DD, *M*=85.3, *SD*=145.7). Seven families dropped-out before study completion, 4 IMM families (2 completed treatment, 2 did not) and 3 WLC families (none completed treatment). Study drop-outs were not statistically different from study completers on relevant baseline variables. Given the small sample size, several events unrelated to treatment (ie, family illness, out-of-state move) had a high impact. In addition, due to the very small budget for this study, participant compensation for completing follow-up assessments was limited. Thus, retention is expected to be better for the proposed study.

Impact on mood. Children improved immediately following treatment, with gains continuing for 12 months post-PEP. Although families were randomly assigned to IMM or WLC, the WLC group had less severe symptoms at baseline. Intervention was associated with improved scores in the IMM group, they approximated WLC means at six and twelve months. The mean MSI for IMM families decreased from severe range (mean = 38.1) to the mild to moderate range (mean = 21) while WLC families began in the middle of the moderate range (mean = 26.4) and at 12 months were in the moderate to mild range (mean = 19.5) of symptoms. Power calculations[69] using $\alpha=.05$ and power=.80 indicate that from baseline to six months, an effect size of .45 was detected, with 64 participants per cell needed to find significance in a larger sample. From baseline to 12 months, an effect size of .60 was detected, with 36 participants per cell needed to detect significance.

Consumer evaluation. Eleven parent (15 item) and 10 child (13 item) evaluations were available for review. Evaluations were positive (parent *M*=1.6, *SD*=0.6; child *M*=1.7, *SD*=0.6 using a Likert scale; 1= strongly agree, 5=strongly disagree). Parents and children reported: 1) learning about symptoms and medications for mood disorders and ways to manage symptoms at home and at school; 2) feeling comfortable with and benefiting from their work with the therapist; and 3) benefiting from the materials and activities provided.

Treatment refinement. Consumer comments, supervision notes from the three study therapists and PI, and a decision to equalize clinician: family face-to-face time between MF-PEP and IF-PEP led to expanding IF-PEP from 16 to 24 sessions. The additional eight sessions cover: healthy habits (2); school-based issues (1); school professional consultation (1); sibling relations (1); and additional "in-the-bank" sessions (3). If a child is not experiencing problems at school or has no siblings, those sessions would be dropped. Also, previous findings suggest not all families will request "in-the-bank" sessions. Thus, the actual number of sessions a family might attend to receive a complete "dose" of IF-PEP ranges from 17-24.

Case series trial of IF-PEP 24. This expanded version of IF-PEP was trialed with two families, both of whom showed improvement following treatment. Three out of four mood scales improved from baseline to post-treatment, and parental report of the child's behavior toward family members demonstrated improvement (more positive and less negative interactions following treatment).[46]

Commercialization project. A small business grant (R41 MH077358) was awarded to the Co-PI (MAF/ Child & Family Psychological Services, Inc.) to develop a commercially available treatment manual for IF-PEP. **The treatment manual is currently in press. Parent and child IF-PEP workbooks and parent, child and child therapist MF-PEP workbooks are also in press[70].**

Design and Method

Sixty children aged 8-12 years with MDD and/or DD and CDRS-R[64] scores ≥ 40 will be randomized to a 2 x 2, **12**-week supplement double-blinded comparison of omega-3 (Ω3) fatty acids vs. placebo (PBO) and IF-PEP vs. active monitoring (AM). **Participants will not be permitted to receive treatment outside of this study protocol beginning one month prior to study participation throughout the entire 12 week trial.** The PBO group will receive capsules matched to the active Ω3 treatment; half of participants randomized to PBO will also get IF-PEP. The AM group will attend assessments, which approximates an attention-control group. Half of participants randomized to AM will also receive Ω3. Importantly, previous studies of childhood depression suggest that simply participating in the repeated assessment of depressive symptoms can lead to symptom improvement.[71] In fact, the American Academy of Pediatrics very strongly recommends that primary care physicians consider a period of 6 to 8 weeks of active monitoring, weekly or biweekly visits, for children with mild to moderate depression prior to recommending antidepressants or psychotherapy treatment.[72] **Fifteen** children will be randomized to each of 4 conditions, Ω3 + IF-PEP, Ω3 +AM, PBO + IF-PEP, PBO + AM. **Seven** assessments will be conducted with each child throughout a **12**-week period. **Participants receiving** IF-PEP **will participate in** 17 to 24 **twice-weekly** therapy sessions**.** The first visit (week -1) will be used to determine study eligibility, including ability to swallow capsules, as described below. Randomization will occur at the second visit (week 0). Main outcomes (CDRS-R, CGI-S/I, and C-GAS scores) will be collected for each group every 2 weeks for the first **6** weeks and every **3** weeks for weeks **6-12**. Blood draws will take place at weeks **0 and 12** to analyze fatty acid plasma levels.

Table 1 *Study Design Summary*

	2 X 2, Supplement Double-Blind<br>Omega-3 (Ω3) vs Placebo (PBO) and<br>Individual Family Psychoeducational Psychotherapy (IF-PEP) vs. Active Monitoring (AM)			
Task	Screen	Randomize<br>Ω3+ IF-PEP, Ω3+AM,<br>PBO+IF-PEP, PBO+AM	**12**-week trial	
			Ongoing Assessment	Endpoint Assessment
Week	-1	0	2, 4, 6, **9**	12
Visit	1	2	3, 4, 5, 6	7
Treatments	No treatment<br>Complete pill swallowing evaluation<br>(& training, ≤ 3 sessions, as needed)	Start Ω3 or PBO<br>And IF-PEP or AM	*n*= **15** Ω3+PEP,<br>*n*= **15** Ω3+AM,<br>*n*= **15** PBO+PEP,<br>*n* = **15** PBO+AM	
Assessments	DSM-IV by P-ChIPS, ChIPS & clinical eval., medical history, vital signs, PE, WASI, Grids, demographics, FHS, EEAC, ATQ, SRCS, UMDQ, CDRS-R, **CDI,** C-GAS	CDRS-R, Grids, vital signs, **CDI,** C-GAS, CGI-S/I, DM, blood draw	CDRS-R, Grids, vital signs, SE, **CDI,** C-GAS, CGI-S/I	CDRS-R, Grids, PE, vital signs, EEAC, SE, ATQ, SRCS, UMDQ, **CDI,** C-GAS, CGI-S/I, DM, blood draw
Duration	4-5 hours	2.5-3 hours	1-1.5 hours	2.5-3 hours

Abbreviations: ATQ=Automatic Thoughts Questionnaire; **CDI=Children's Depression Inventory;** C-GAS=Children's Global Assessment Scale; CGI-S/I=Clinical Global Impressions Severity & Improvement Scales; ChIPS=Children's Interview for Psychiatric Syndromes P-ChIPS=parent version; DM=Dietary Monitoring; FHS=Family History Screen; Grids=Medication Usage and Services Grids; PE=Physical Exam+ Anthropometric measures; SE=Side Effects Review; SRCS=Self-Report of Coping Skills; UMDQ=Understanding Mood Disorders Questionnaire; WASI=Wechsler Abbreviated Scales of Intelligence

Participants

Sample. **Sixty** children aged 8-12 years will be enrolled at the rate of **2.5** per month. We have constructed an extensive referral network and are able to generate a participant pool with diverse ethnic and socioeconomic backgrounds (see Recruitment in the Human Subjects section).

Inclusion criteria: 1) aged 8-12 years (boys and girls); 2) DSM-IV-TR[73] diagnosis of MDD and/or DD as determined by consensus conference (described below); **2)** CDRS-R score ≥ 40; **3)** full scale IQ ≥ 70; **4) child** and at least one parent must be able to complete all assessment; **5) child** must be able to swallow capsules (training in swallowing will be offered)**; 6) parent and child must be willing to have blood drawn from child at visits 2 and 7.**

Exclusion criteria. Participants must not have: 1) major medical disorders (eg diabetes, epilepsy, metabolic disorder); 2) inability to communicate in English; 3) lack of access via phone; 4) autism; 5) psychosis; **6) DSM-IV-TR[73] diagnosis of a bipolar disorder by consensus conference (described below); 7)** active suicidal concern (e.g., "I want to kill myself", a plan for suicide, or an attempt in the past month; **however, passive suicidal ideation, such as "I wish I were dead" would not exclude**); 8) intake in the previous 4 weeks of supplemental Ω3 fatty acids.

Recruitment

Our previously developed referral network includes local primary care physicians and mental health professionals. **Also**, Drs. Fristad and Arnold are currently following children aged 6 to 12 in LAMS (R01 MH073801-06, Longitudinal Assessment of Manic Symptoms). We anticipate a number of these children, 14% (n=25) of whom have MDD and/or DD, and their families will take interest in participating.

Enhancing recruitment is our recently integrated Ohio State University-Nationwide Children's Hospital (OSU-NCH) Division of Child & Adolescent Psychiatry. NCH-Behavioral Health Clinics (NCH-BH) have four community-based settings with over 100,000 outpatient visits annually. Additionally, the OSU Outpatient clinic has over 6,000 visits annually. In the LAMS study, we enrolled 185 youth with elevated symptoms of mania (i.e., scored ≥ 12 on the General Behavior Inventory Short-Form: GBI-SF, 12% of the sample were screen negatives)[74] in a 2.5-year time period (approximately 6/month). The NCH-BH intake system was recently reorganized, one goal of which was to facilitate provision of information about research treatment opportunities to families prior to their intake evaluation. **This should** further enhance recruitment. Minority recruitment will be enhanced by a four-pronged plan that includes personalized contact with and distribution of recruitment materials to key minority leaders (church leaders, physicians, community leaders) **(see Human Subjects section).**

Attrition

We do not know what attrition will occur. This, in fact, is one purpose of the study: to determine feasibility of recruitment **and** retention for a larger, more definitive study if this pilot study suggests Ω3 and/or IF-PEP offers some clinical benefit. Of the **three** clinical trials of **DHA/EPA** Ω3 and mood in children known to the researchers, the average attrition rate **was 22**%; **three** drop-outs **(4.5% of participants)** were due to side effects **(GI disturbance)**[25, 26, 27, 28]. This is **comparable** to a 30% drop out rate in pharmacologic clinical trials of depression and bipolar disorder due to adverse events, lack of symptom improvement, or both.[75] In our large-scale RCT, only 12% of those in immediate treatment did not complete MF-PEP and in our RCT of IF-PEP, 20% in immediate treatment did not complete IF-PEP. There is always the possibility in this study that participants will drop out and purchase Ω3 supplements on their own or get tired of taking them. Similarly, families may decide they are not interested in IF-PEP therapy sessions and will terminate those early. However, both Co-PIs have experience in minimizing attrition in clinical trials of nonpharmacological treatments. In fact, Co-PI Arnold has recently completed a sample of 52 in a double-blind study of 21 weeks total duration with similar visit frequency for a different supplement and experienced only **1** dropout during the double-blind phase. Frequent contact by a well-trained staff and access as needed to Drs. Fristad and Arnold by study participants are among the strategies expected to minimize attrition. In our previous experience, increasing visit frequency from every 2 months to every 2 weeks in a nutritional trial decreased attrition from about 25% to <3%. Visit frequency will be every two weeks for weeks **0 to 6** and every **three** weeks for weeks **6 through 12**. If a family terminates the study early, every effort will be made to ascertain the reasons for their departure, which will be documented on the Early Termination Form (see Appendix B) administered via phone interview or self-report, based on the family's willingness to share information. Differential attrition between Ω3/placebo and IF-PEP/active monitoring and will be analyzed as an additional palatability/feasibility outcome as well as possible indicator of satisfaction with treatment results. Attrition will be used as one measure of feasibility and will be used to estimate the number of participants needed in a larger trial.

Adherence

Participant adherence will be checked by standard **capsule** counts of returned unused dosage forms. Data will be recorded on a Medication Accountability Form (see Appendix C). Adherence below 86% (1 missed-dose day per week) will result in re-instruction and emphasis on the importance of taking the capsules regularly. Adherence below 70% (2 missed days/week) without good reason (such as gastroenteritis) will result in assistance with planning where to keep the doses, when to take them, and how to remember. Participants will be given pill minder boxes and capsule-administration logs to aid in adherence. All participants will be retained regardless of adherence as long as they are willing to be assessed. Differential rates of study drop-out will be examined as an outcome (see Hypothesis #2). Palatability and adherence have great practical importance both for clinical use and for the large randomized clinical trial we hope will follow this pilot study.

Ω3 Dosage and EPA:DHA Ratio

Definitive guidelines re: dosage and EPA:DHA ratio are lacking. Adult studies tend to use 1-2 g with widely varying EPA:DHA ratios. Studies of Ω3 in children vary widely in both their dose and EPA:DHA ratio (560-8075mg EPA+DHA; 7EPA:1DHA to 1EPA:4.3DHA) Thus, we determined to test a mid-range dose and a ratio that has received some past scrutiny. **The Ω3 group will receive 1000 mg Ω3 (two 500 mg capsules, each containing 350 mg EPA: 50 mg DHA; 100 other Ω3) two times daily for a total daily dose of 2000 mg Ω3 (1400 mg EPA: 200 mg DHA; 400 other Ω3). The placebo group will receive two capsules two times daily matched for odor and appearance with the active intervention.** Ω3 and placebo will be provided by OmegaBrite (www.omegabrite.com; see letter of support). We will provide all participants with a multi-vitamin/mineral to control for vitamin and mineral levels, **but no** other nutritional supplements will be allowed.

Concomitant Treatment

Participants will be instructed not to receive treatment for depression outside of this study protocol beginning one month prior to study participation throughout the entire 12 week trial, unless deemed necessary by their physician or through referral of the co-PIs due to medical necessity. **If potential participants have received outside treatment within the month prior to screening but are unsatisfied with this treatment, they may be randomized once they have been treatment-free for at least a month. *No participant will have satisfactory current treatment stopped or altered* to participate in this study.**

Procedures

The Co-PIs and post-doctoral Study Coordinator will conduct initial evaluations with primary parental informants and children, and the graduate research associate (GRA) will conduct subsequent assessments. The post-doctoral Study Coordinator will provide individual IF-PEP sessions to participants in the Ω3+PEP and PBO+PEP groups. Two of the investigators, a physician and a research pharmacist will monitor dosages and distribution of all supplement capsules. A co-investigator is a nutritionist and will review nutritional health information for general safety purposes. Visits at Weeks **0 and 12** will be scheduled in the morning to accommodate fasting blood draws. The child will be provided breakfast following the blood draw.

Phone pre-screening. The **phone** pre-screening procedure is designed to address the following questions: Does the child have a high likelihood of meeting current DSM-IV diagnostic criteria for MDD and/or DD? Does the child have a diagnosed metabolic disorder or other chronic health condition? Does the child attend regular classes? (Clarifying questions will be added, as needed, to determine if the child has a low IQ.) Does the child live with one or more parents/caregivers? Is it likely that the child and one or more parent(s)/ caregiver(s) will be interested in participating in the research protocol (after hearing a brief description of it)? If this prescreening interview indicates possible interest in and eligibility to participate in the study, the child and parent(s)/caregiver(s) will be invited to a screening appointment, at which time informed assent and consent will be obtained and the screen assessment completed. Parents of ineligible children will be offered referral information to seek other mental health services as appropriate.

Screening. Children and their parent(s)/caregiver(s) will provide written informed assent/consent and authorization to use personal health information in research (HIPAA), then complete screening assessment materials. Children's pill swallowing ability will be determined at this time. Children who can swallow **capsules** the size used in the study at screening will require no further intervention. Children unable to swallow pills will proceed with the desensitization protocol (see swallow-training protocol in Appendix D). Up to 3 visits can be scheduled, if needed, for clinical supervision of the desensitization, and/or their parents can work with them at home to complete the desensitization. Children who are able to swallow study-sized pills **and have not received depression treatment for ≥ 1 month** can be scheduled for their randomization visit within a week. Children not capable of swallowing study-sized pills even after 3 training sessions will not be randomized: the number/percentage of such children initially recruited will be noted and reported as a feasibility measure.

Randomization. At the randomization visit, children will receive their random assignment to one of four conditions (Ω3+PEP, Ω3+AM, PBO+PEP, and PBO+AM). We will block-randomize in blocks of 4 (Ω3+PEP, Ω3+AM, PBO+PEP, and PBO+AM). While it would be desirable to balance on (a) primary diagnosis (ie MDD **vs**. DD), and (b) comorbid conditions (ie, behavior, anxiety and other disorders), this pilot sample size of **60** does not allow such a strategy.

Children and their parents will be educated, based on their randomization assignment, to the appropriate regimen(s) (see Table 3 below). Children will come to the clinic for medical, nutritional and symptom evaluation every other week for the first **6** weeks and **every three weeks for weeks 6 to 12**. Children who are assigned to the IF-PEP condition will schedule **twice-weekly** therapy sessions with the SC.

Blindness of Evaluators. One full-time post-doctoral study coordinator (SC) and one half-time graduate research associate (GRA) enrolled in the OSU doctoral program in clinical child psychology will conduct interviews with parents and children at screen. To maintain the blind, the GRA will con-

duct baseline and follow-up evaluations as the SC will be providing therapy and thus no longer blinded to the IF-PEP condition. While Dr. Fristad can provide general supervision of the GRA, Dr. Arnold will provide ongoing supervision of CDRS-R, C-GAS and CGI-S/I ratings as Dr. Fristad will be unblinded by supervising the SC who is providing IF-PEP. A research pharmacist not involved in assessments will dispense all Ω3 and matched placebo without un-blinding the GRA. Participants will remain blinded with placebo matched by size, color, shape, and taste.

Study time frame. Three years will be required to complete the study. The first three months will be spent hiring and training staff, preparing pharmacy packages, and initiating recruitment strategies. Enrollment will occur from month 4 to month **27**. All participants will complete all assessments by month **30**, leaving six months for data analysis and manuscript preparation (see Table 2).

Table 2 *Study Schedule*

--------------Year 1----------------*----------Year 2------*----------------------------Year 3---------------------------------

Mos. 1-3	*Mos.* ***4-27***	*Mos.* ***28****-30*	*Mos. 31-36*
Hire & train staff, operationalize study	Enroll **2.5** Ss/month, conduct assessments and treatment	Complete all treatment and assessments	Analyze data, prepare manuscripts & submit R01

Study coordination. Comprehensive research team meetings will be held weekly initially, then at least **every other week.** These meetings will provide a forum for the entire multidisciplinary team to discuss methodological issues related to assessment, intervention, data management and analysis. **No information that could un-blind the GRA will be discussed while the GRA is present.**

Instruments. **See Appendix E for descriptions of each instrument used in this research plan.**

Consensus diagnosis and ongoing ratings. The assessment team (Drs. Fristad and/or Arnold, the post-doctoral Study Coordinator and the psychology graduate research associate [GRA]) will meet within **2 business days** of screening assessments prior to randomization. During these meetings, study eligibility decisions will be finalized and consensus diagnoses will be determined for participants. Consensus diagnoses will utilize all information obtained. Data from ChIPS/ P-ChIPS will **follow the "either-or" rule** (ie, counting a symptom as positive if either the child or parent endorses it) unless there are clear and compelling reasons to disregard information (eg, if the child responded "yes" to everything asked; if the parent/caregiver exaggerated **all answers**).

Nutritional Assessments

These will be conducted at Weeks **0 and 12** at the OSU Clinical Research Center (CRC) (a 5-minute walk from the OSU Childhood Mood Disorders Lab). Blood draws will be completed by research nurses trained in pediatric blood draws and nutrition assessments by research dieticians highly experienced in these procedures (see letter of support). Dr. Belury will direct the fatty acid assays.

Assays of fatty acid levels. To determine the extent that participants consumed and accumulated Ω3 fatty acids, we will analyze non-esterified fatty acids by gas chromatography of plasma levels of EPA and DHA. As we have shown[48], plasma fatty acid composition reflects fatty acid intake of the past few days to week of supplementation with Ω3 supplements. Therefore, plasma fatty acid analysis should reflect, in addition to **participant-reported** adherence, actual intake of supplements over several days prior to each **blood test**. In a recent pilot study (as part of the NCI-NIH HER-2/neu and dietary fat: **gene-nutrient** interactions in breast cancer grant for which Dr. Belury is Co-Investigator), we found a significant accumulation of EPA and DHA in plasma within one month in adults receiving approximately equivalent doses (expressed as Ω3/kg body weight) compared to **doses** proposed here for children. Levels remain elevated in plasma for up to 6 months if participants continue consuming supplements. **Our** preliminary findings in adults demonstrate using the dose proposed in the present study will likely result in an approximate two-fold increase of EPA and DHA levels.

Fatty acid composition will be analyzed using a two-step procedure described previously.[76] Total lipids will be extracted using the method of Bligh and Dyer.[77] Fatty acid methyl esters (FAMEs) will be prepared from each lipid fraction by incubating samples with tetramethylguanidine at 95°C[78] and quantified using a Hewlett Packard 5890 gas chromatograph equipped with an auto-sampler ChemStation software (Agilent Technologies, Meriden, CT), FAME ionization detector and a 30-m Omegawax 320 capillary column (Supelco Co.).[48] Helium flow rate will be 30 ml/min and oven temperature will be programmed to start at 175°C then ramped to 220°C at 3°C per minute. Identification of FAMEs will be accomplished by comparing retention times of samples to retention times of authentic standards (Supelco Co.). The internal standard heptadecanoate (17:0) will also be used to quantify absolute amount of fatty acids in ng/ml. Dr. Martha Belury (Co-I) will direct these analyses. **Besides** these specialized assays, routine cholesterol fractions and triglycerides will be checked.

Dietary monitoring. Diet will be monitored with the standardized USDA Automated Multiple Pass Method[79] in addition to Food Frequency Questionnaires (FFQs). Using a 5-step multiple-pass method, children and parents will be interviewed about the child's food/beverage consumption for the previous 24-hour period.[80,]

[81] The 24 Hour Recall or Typical Diet Form (see Appendix F), supplemented by samples of plastic and paper food models and portion estimates, will be used to increase precision in the data gathered. This method reduces underreporting of intake due to repeated opportunities to recall foods and beverages.[79]

Dietary records will be entered into the Nutrition Data System for Research (NDS-R) software by a research dietician.[82, 83] **This** nutrient analysis program **is** maintained by the Nutrition Coordinating Center (NCC) at the University of Minnesota's School of Public Health. The NCC maintains a database containing 139 nutrients, nutrient ratios, and food components. The NDS-R software provides data for 18,000 foods and 8,000 brand names of foods. The NCC database is derived from the USDA database, scientific literature, food manufacturers and foreign food composition tables. Individual and group data and comparisons to national recommendations can be exported into spreadsheet or database analysis programs for further statistical comparisons. Food groupings can be reviewed and scored according to the method developed by Kant[84] using the data generated by NDS-R to determine if diet pattern has changed over the study. Likewise, the diversity score can be determined by the number of food groups consumed daily[85] and compared to food groups recommended by the Dietary Guidelines for Americans.[82, 83, 86, 87]

Interviewers

Non-nutrition assessments will be conducted by project personnel hired for this study. One full-time post-doctoral study coordinator (SC) and one half-time graduate research associate (GRA) enrolled in the OSU doctoral program in clinical child psychology will conduct interviews with parents and children respectively at screen. To maintain the blind, the GRA will conduct follow-up evaluations as the SC will be providing therapy and thus no longer blinded to the therapy treatment condition. While Dr. Fristad can provide general supervision of the GRA, Dr. Arnold will provide ongoing supervision of CDRS-R, C-GAS and CGI-S/I ratings as Dr. Fristad will be supervising the SC who is providing IF-PEP, and thus, will not be blind to psychotherapy assignment. Ideally, the same staff will remain on the study for its duration, allowing for continuity of assessors. An interviewer can complete four interviews, on average, per week, including all requisite form completion, given the standard rate of no-shows and cancellations. Thus, interviewing staff will also be able to oversee various project tasks to maximize their time management. Interviewers will go through a detailed training program led by the Co-PI (MAF) prior to conducting interviews. The training program will include extensive instructions on child psychiatric syndromes; how to administer questionnaires; information regarding clinical and ethical issues involved in interviewing; practicing, observing and rating interviews; performing mock interviews and receiving feedback from experienced study personnel; and undergoing reliability checks. Project personnel will observe and concurrently rate mock interviews. When new interviewers have achieved reliability on all administered interviews (*kappas* $\geq$ .70) they will be videotaped conducting live interviews. Videotaped interviews will be reviewed by Drs. Fristad and Arnold. After reliability is achieved in live interviews (*kappas* $\geq$.70), project personnel can interview independently. All families will be asked to consent to being videotaped. Ten percent of tapes will be randomly chosen to be reviewed and independently rated. If these checks uncover rater "drift", that interviewer will be required to repeat training procedures until the reliability standard (*kappas* $\geq$ .70) is regained. Approximately one hour per week of on-site supervision will be provided on a weekly basis. Our Multi-family Psychoeducation Group (MF-PEP) study experience has taught us that kappas are actually above .70 once proper training is completed, and ongoing supervision appears to successfully assist in maintaining high levels of reliability (range for ChIPS, P-ChIPS and mood rating scales: *kappa* = .70-.85).

Therapist

The SC will conduct all IF-PEP treatment with families enrolled in the study. S/he will have completed doctoral training and an internship in clinical child psychology, be trained by the Co-PI (MAF) in conducting IF-PEP and receive weekly individual supervision from the Co-PI to ensure treatment adherence and address any questions or concerns raised by participating families or the therapist. A treatment adherence scale currently being developed by the Co-PI will be used in this process.

Participant Reimbursement. In recognition of travel costs, other expenses, time (possibly lost time from work), and inconvenience, the child and parent will be paid upon completion of each assessment. **See payment schedule in Human Subjects Section.**

Potential Problems/Limitations and Alternative Strategies Considered

We considered studying only one intervention in this trial; however, TADS[6] provides compelling rationale for studying combination treatment **in adolescent depression and a series of other studies have indicated superiority of combined treatment for other disorders (PDD, anxiety, ADHD)**. Additionally, the 2X2 design allows us to examine the potential efficacy of each intervention separately, as well as together.

Data Analysis

The data analytic plan has been developed in conjunction with Dr. William Gardner, a developmental-quantitative psychologist. Dr. Gardner will plan and supervise analyses in collaboration with the Research Team. It is recognized in this pilot study that we will focus primarily on determining feasibility and potential effect sizes. Only secondarily will we conduct exploratory examinations of the data.

Sample size determination. Due to the exploratory nature of the pilot study, the sample size of **60** was chosen purely for practical reasons of recruitment and time and monetary limitations of an R34 grant. The 2X2 design (**30** participants per each active treatment; **15** with each combination [Ω3+PEP, PBO+PEP, Ω3+AM, PBO+AM]) grants more power to examine potential moderators of treatment response to Ω3. This proposed pilot study should provide necessary information for planning a large-scale, definitive study. Negative findings in the pilot study might be attributable to insufficient power to detect meaningful effects with high confidence; in a sufficiently powered study, a negative finding is more conclusive. While the proposed pilot study is not being used to determine an effect size for a subsequent large scale study, this pilot study will provide a check to see if the desired effect size ($d \geq 0.5$) is plausible.

Operationalized variables. The primary outcome measure is the CDRS-R. Additional outcome measures include **CDI,** C-GAS, CGI-S & CGI-I. Potential mediators include: *Adherence*--% of pills consumed, EPA and DHA levels, and % of IF-PEP sessions attended; *Negative Automatic Thoughts*—ATQ; *Coping Strategies--SRCS; Knowledge of Mood Disorders*—UMDQ; *Family climate*—EEAC*.* Potential moderators include: *baseline EPA and DHA levels, family history of mental illness-* Family History Screen; *Children's Axis I Comorbid Disorders:* Consensus diagnoses of behavioral, anxiety and 'other" disorders, as derived from the ChIPS or P-ChIPS; *Psychosocial stressors*—as obtained on the ChIPS and P-ChIPS. **Treatment history** and subsequent changes in **treatment** will be quantified using the Medication and Service Providers Grids. Side-effects will be monitored using the Side Effects Review.

Exploratory data analysis and missing data. Exploratory data techniques will be used to examine all distributions for outlying or aberrant observations.

Intent to treat (ITT). All participants will be analyzed as members of the group to which they were randomized, regardless of actual adherence with the treatment regimen (as defined by pill counts and number of IF-PEP sessions attended).

Model. A linear mixed effects model (LME)[88] will be fit to each outcome variable. The random effects are intercept and slope; which posit that each participant has a "personalized" linear response to treatment. The fixed effects are treatment X time interactions, which measure systematic differences in the rate of change. The assumption of a common initial mean for all participants is justified by the randomization of participants into treatment groups. Here an additional term for random "noise" allows for measurement/replication error. A major advantage of the LME model is that it does not require a balanced design; in particular, participant effects can be estimated using incomplete data. Thus there is no need for imputation as long as missing data are missing at random.[89]

Table 3. *Summary Table—Treatment Groups*

1. PBO+AM	2. PBO+PEP	3. Ω3+AM	4. Ω3+PEP

Major hypothesis testing.

1. *Feasibility--Participants can be recruited in* ***a two–year period****.* Descriptive statistics will be used to characterize the recruited sample **and time frame. All 4 groups (1-4)**
2. *Attrition in PBO+AM vs other 3 groups at Week* ***12***: We will use negative binomial regressions[89] to analyze these data. **Group** 1 vs 2/3/4
3. *Ω3 will have a clinically meaningful effect size on depressive symptoms reported on the CDRS-R compared to placebo at Week* ***12***: The effect size of interest is the differential rate of change attributable to Ω3 versus placebo, expressed as relative to the (assumed) common standard deviation of individual rates within group. Restricted maximum likelihood estimation (REML) will be used to reduce bias when estimating the between and within group variance components of the random slopes (rates of change). The effect size is estimated as $d = \left(\bar{b}_t - \bar{b}_c\right) / s_b$, where $\bar{b}_t$ and $\bar{b}_c$ are the mean rates of change in the treatment and control groups respectively, and s_b is the pooled within group standard deviation of the individual slopes. If $d \geq .5$, that will be considered *prime facie* evidence for proposing a large-scale investigation. **(However, the effect size found will not drive the power analysis for reasons elucidated by Kraemer).** As a secondary means of assessing change, chi square analyses will be used to determine the relationship between response (as defined above) and treatment status (PBO vs Ω3). 1/2 vs 3/4

4. *PEP will have a clinically meaningful effect size **(d ≥.5)** on depressive symptoms reported on the CDRS-R compared to active monitoring at Week 24*: this will be analyzed in the same manner as #2 above (PEP vs. AM). 1/3 vs 2/4
5. *Combined treatment (Ω3+PEP) will demonstrate a clinically meaningful additive effect (d≥.25) on depressive symptoms reported on the CDRS-R compared to either individual treatment (Ω3,IF-PEP) at Week* ***12***. Comparisons will be made as described above. 4 vs 2, 3

Secondary analyses.

1. To explore rates of change and timing of clinical response we will graph clinical responses to Ω3 and IF-PEP on the CDRS-R and CGI-S/I to see if and how soon any benefit is noted and when it plateaus. Main outcome variables will be plotted in ITT graphs where the horizontal (ie, time) axis spans the entire **12** week duration of the study. We will examine whether improvement is sustained (constant rate of change), plateaus or attenuates (decelerating improvement) as the study progresses. Ω3, 3/4; IF-PEP, 2/4
2. To examine clinical significance more broadly, hypotheses #3-5 will be examined via graphs of a variety of outcome measures, including global functioning (C-GAS). Measures that appear particularly sensitive to change will be considered for any larger subsequent study; others will be dropped to minimize participant burden. Same as Hypotheses 3-5 above
3. To explore possible mediating effects, we will examine graphs of the associations between rate of change in clinical outcome and changes in EPA and DHA blood levels, number of IF-PEP sessions attended, and changes in negative automatic thoughts (ATQ), coping strategies (SRCS), knowledge of mood disorders (UMDQ), and family climate (EEAC). Ω3, 3/4;IF-PEP, 2/4
4. To explore possible moderators of outcome, we will examine graphs of the associations between rate of change in clinical outcome and baseline lipid blood levels, comorbid disorders (ascertained on ChIPS and P-ChIPS), psychosocial stressors (ascertained on ChIPS and P-ChIPS), family history of mental disorders (FHS), and treatment history (Service and Med grids). 1-4
5. To track adherence over time, parent- and child self-reported rates of capsule ingestion will be tabulated against **staff** pill counts. Then both self-reported rates of capsule ingestion and pill counts will be correlated with EPA and DHA blood levels after **12** weeks **to check validity of the former**. 3-4
6. Changes in EPA and DHA blood levels will correlate with **changes in** depression ratings (as measured by the CDRS-R) at $r \geq 0.5$.
7. Determine rates of **previous** treatment utilization and side-effects for all participants (as measured by the Medication and Services Grids and Side Effects Review, respectively).
